# A Role for Visual Memory in Vocabulary Development: A Systematic Review and Meta-Analysis

**DOI:** 10.1007/s11065-022-09561-4

**Published:** 2022-09-22

**Authors:** Hayley E. Pickering, Jessica L. Peters, Sheila G. Crewther

**Affiliations:** 1 Department of Psychology, Counselling, and Therapy, La Trobe University, Kingsbury Drive, Melbourne, VIC 3086 Australia; 2 Centre for Human Psychopharmacology, Swinburne University of Technology, Hawthorn, VIC 3122 Australia

**Keywords:** Systematic review, Meta-analysis, Vocabulary, Visual memory

## Abstract

Although attention and early associative learning in preverbal children is predominantly driven by rapid eye-movements in response to moving visual stimuli and sounds/words (e.g., associating the word “bottle” with the object), the literature examining the role of visual attention and memory in ongoing vocabulary development across childhood is limited. Thus, this systematic review and meta-analysis examined the association between visual memory and vocabulary development, including moderators such as age and task selection, in neurotypical children aged 2-to-12 years, from the brain-based perspective of cognitive neuroscience. Visual memory tasks were classified according to the visual characteristics of the stimuli and the neural networks known to preferentially process such information, including consideration of the distinction between the ventral visual stream (processing more static visuo-perceptual details, such as form or colour) and the more dynamic dorsal visual stream (processing spatial temporal action-driven information). Final classifications included spatio-temporal span tasks, visuo-perceptual or spatial concurrent array tasks, and executive judgment tasks. Visuo-perceptual concurrent array tasks, reliant on ventral stream processing, were moderately associated with vocabulary, while tasks measuring spatio-temporal spans, associated with dorsal stream processing, and executive judgment tasks (central executive), showed only weak correlations with vocabulary. These findings have important implications for health professionals and researchers interested in language, as they advocate for the development of more targeted language learning interventions that include specific and relevant aspects of visual processing and memory, such as ventral stream visuo-perceptual details (i.e., shape or colour).

**Supplementary Information:**

The online version contains supplementary material available at 10.1007/s11065-022-09561-4.

## Introduction

Each year in Australia, around 20% of children starting school are identified as having additional learning needs (Goldfeld et al., 2012; O’Connor et al., 2020), with approximately half of this cohort identified as showing speech or language difficulties (O'Connor et al., 2019). Unfortunately, such language difficulties during childhood are known to increase the risk of poor social, emotional, academic, and occupational outcomes throughout the lifespan (Hentges et al., 2021; McKean et al., 2017; Whitehouse et al., 2009). Thus, it is important to understand the early mechanisms of both typical and atypical language development in the brain, so that appropriate learning supports and interventions can be implemented for affected children.

Language is multifaceted, but is best defined as a system of shared signs, symbols, or semantically related sounds, usually verbalizable words termed vocabulary, for the purpose of communication or self-expression (Hoff, 2014; National Institute of Mental Health, 2018). Vocabulary is considered a core aspect of language and is a key marker of overall verbal language development and verbal intelligence, with typical school children learning 10–20 new words each day (Anglin et al., 1993; Trautwein & Schroeder, 2017). Vocabulary encompasses both the words an individual can understand (receptive vocabulary) and the words they can produce and use (expressive vocabulary) (Conti-Ramsden & Durkin, 2012; Deldar et al., 2021). In childhood, vocabulary is typically assessed via visual tasks (i.e., picture naming or matching a spoken word to a picture), and predominately considers knowledge of concrete nouns and verbs. Whilst a range of psychosocial factors are known to influence verbal language and vocabulary development (e.g., male gender, parent-child interactions, and maternal education, vocabulary, and mental health; Eadie et al., 2022; Mous et al., 2017), the cognitive skills and underlying brain mechanisms that contribute to vocabulary development remain largely undefined (Samuelson, 2021).

Memory, particularly short-term memory (briefly holding information in mind; Baddeley, 2012) and working memory (online processing and manipulation of information; Adams et al., 2018), has long been the core cognitive skill implicated in supporting vocabulary development (Baddeley, 2003; Gathercole & Baddeley, 1989). Research techniques examining the contribution of memory to vocabulary development vary with the age of the child and expected verbal skills. For instance, research in infancy and early childhood (up to 3-years of age) has primarily focused on visual memory contributions to early language and vocabulary, including joint attention, visual working memory, and spatial recall (Cetincelik et al., 2021; Mundy et al., 2003; Ortiz-Mantilla et al., 2008; Samuelson, 2021; Yu et al., 2019). In contrast, research in preschool and school-aged children has instead focused almost exclusively on auditory-verbal memory contributions to ongoing vocabulary development (Baddeley, 2003; Baddeley et al., 1998; Bowey, 2001; Metsala, 1999; Rispens & Baker, 2012; Verhagen et al., 2019). Thus, potential contributions of visual memory processes (alternatively termed visuospatial memory processes) to vocabulary development have seldom been investigated beyond infancy and early childhood. As such, our current understanding of the nature of this association and its potential ongoing importance beyond these early years remains unclear. This is concerning given many aids, strategies, and interventions employed to help children with language difficulties are often visually-based (Steele & Mills, 2011), yet how these visual aids are utilised by children, and how best to implement them, remains relatively under investigated. Furthermore, understanding of the possible association between visual memory and vocabulary development has been hindered by the variability and inconsistency in the types of visual memory tasks used in the literature, and in the theoretical interpretation of what these tasks measure. We propose that interpretation of these tasks and associated findings would likely be improved by using a brain-based understanding of the neuroanatomical networks associated with visual sensory processing, given these networks have been shown to support visual attention and memory processes (D'Esposito, 2007; Eriksson et al., 2015; Wager & Smith, 2003). Hence this review aimed to systematically investigate current literature examining the association between visual memory and vocabulary across childhood (2- to 12-years), with a focus on how a neuroscientific knowledge of visual processing and memory mechanisms in the brain may better inform understanding of this association.

### Vocabulary Growth Throughout Childhood

As already alluded to, language research in infancy and early childhood emphasises the importance of visual cognition and memory processes to vocabulary development. For instance, a recent systematic review by Cetincelik et al. (2021) highlighted the central role of eye gaze, fixation, and joint attention (shared focus between two individuals) in early social communication. Indeed, effective and efficient ‘looking’ (i.e., short, focused fixations) has been shown to enhance visual working memory, support word-object mappings, and is predictive of vocabulary abilities at 12- to 18-months (Cetincelik et al., 2021; Gregory & Jackson, 2016). Additional visual processes, such as spatial and sustained attention, processing speed, and visual working memory, have also been shown to facilitate infants’ language learning by supporting object recognition and processing of object-word mappings (Samuelson, 2021; Yu et al., 2019). Infant multisensory association skills (i.e., mapping a spoken word to a visual object) have also been linked to higher receptive and expressive vocabulary skills in toddlerhood (Cetincelik et al., 2021; Choudhury et al., 2007). Beyond infancy, children have been shown to use a range of visual skills to support ongoing vocabulary learning. For example, pre-schoolers (4-6-years) who are taught new words with pictorial support learn (and retain) the words better than those learning without pictorial support (Strauber et al., 2020), older children (7-13-years) learn word lists better when the information is presented as pictures, or pictures and words, rather than as spoken words alone (Constantinidou & Evripidou, 2012; Constantinidou et al., 2011), and children gain vocabulary through reading (Wasik et al., 2016). Thus, there is evidence that visually acquired information retains an important role in vocabulary learning across childhood, although the specific contribution of visual memory has rarely been evaluated.

Indeed, research regarding vocabulary development in older children (4-years and above), has instead largely focused on auditory-verbal memory impairments in children with language delays or impairments. Such children, typically referred to as having a Developmental Language Disorder (DLD), or previously Specific Language Impairment (SLI), have impaired development of their first language compared to neurotypical peers, despite adequate hearing and opportunity for language learning (Bishop et al., 2017). Children with DLD can present with impairments in a range of language domains, including grammar, syntax, and, of interest here, vocabulary (Bishop, 2006; McGregor et al., 2013), with around 7% of school-aged children meeting criteria for DLD (Norbury et al., 2016; Tomblin et al., 1997). Whilst there is consensus in the literature that DLD children have impaired auditory-verbal short-term and working memory (relative to their neurotypical peers; Archibald & Gathercole, 2006a; Vugs et al., 2013), evidence regarding visual memory impairment is mixed. In a meta-analysis of visual memory impairment in DLD/SLI children, Vugs et al. (2013) found moderate effect sizes for both visual storage memory tasks (indexing short-term memory, *d* = 0.49 [0.30–0.68]) and visual central executive memory tasks (indexing working memory, *d* = 0.63 [0.27–0.99]), suggesting the DLD/SLI children performed worse than age-matched neurotypical peers on such tasks. However, heterogeneity analyses indicated substantial variability in these results (*I*^2^ = 50–67%), which could not be accounted for by age or the severity of language impairment (language impairment in a single domain versus language impairment in multiple domains), suggesting other factors may moderate whether DLD/SLI children show impairment on visual memory tasks. Further, in studies that have included both DLD and neurotypical children, correlations between visual memory tasks (including spatio-temporal block tapping tasks, spatial array recall tasks, and recall of visuo-perceptual details) and vocabulary range considerably, from very weak to moderate (r = –.04–.54; Henry & Maclean, 2003; Lum et al., 2012; Vugs et al., 2017), indicating more research is needed to understand what is driving such variability. Visual memory also appears to be more specifically associated with receptive vocabulary (as compared to expressive vocabulary; Nickisch & Von Kries, 2009). Together, such results suggest an association between visual memory and language/vocabulary in both DLD and neurotypical children. However, the substantial variability in findings means that our understanding of how visual memory may contribute to vocabulary development, including which aspects of visual memory are most related to vocabulary, remains unclear and requires further investigation.

## Language and Visual Processing in the Brain

To better understand how visual memory could contribute to vocabulary development, it is important to consider the neural networks underlying visual and language information processing in the brain. From birth, attention to any incoming sensory information in young primates, including human infants, is primarily visually directed via eye movements (Bullier, 2001; Mundinano et al., 2019). Eye movements are predominantly driven via the fastest retinal pathways to the thalamus, superior colliculus, and parietal cortex (Laycock et al., 2020), with aspects of the visual scene processed by the major dorsal and ventral cortical streams (Goodale & Milner, 1992). The dorsal occipito-parietal-frontal “vision for action” or “where” pathway is preferentially associated with the rapid dynamic control and direction of visual attention and processing of spatial and temporal information (Corbetta & Shulman, 2002; Kravitz et al., 2011). Alongside this, the ventral occipito-temporal “vision for perception” or “what” pathway is known to preferentially processes visuo-perceptual details (e.g., object, shape, colour) (Goodale & Milner, 1992; Kravitz et al., 2013).

The importance of differentiating dorsal (spatial) and ventral (visuo-perceptual) processing for visual memory is evident from brain imaging studies showing that these fundamental sensory processing pathways support information maintenance during working memory tasks (e.g., with the dorsal stream activated via spatial information during spatial working memory tasks) (D'Esposito, 2007; Eriksson et al., 2015; Wager & Smith, 2003). Behavioural studies also support a distinction between the processing of spatial and visuo-perceptual memory details (Pickering et al., 2001; Simmering et al., 2015). Evidence from brain imaging studies of language processing demonstrate that occipitotemporal areas related to the ventral visual pathway (including the temporal pole, inferior and superior temporal gyrus, and fusiform gyrus) also support language processes such as object naming and semantic association (i.e., word meanings; Binder et al., 2009; Deldar et al., 2021; D'Esposito, 2007; Ellis et al., 2006). Indeed, a recent review from Deldar et al. (2021) has highlighted a significant overlap between core language areas of the brain and established working memory networks.

We suggest that application of this neural network approach to understanding visual processing may reduce much of the variability found in past research relating to DLD children. For instance, most previous studies examining spatio-temporal block tapping tasks or spatial arrays (requiring dorsal visual stream processing) reported no differences between DLD children and neurotypical age-matched controls (Archibald & Gathercole, 2006b; Arslan et al., 2020; Botting et al., 2013; Briscoe & Rankin, 2009; Hutchinson et al., 2012; Lukács et al., 2016; Lum et al., 2012; Petruccelli et al., 2012; Vugs et al., 2017; Williams et al., 2000; although see Bavin et al., 2005, and Hick et al., 2005a, b for evidence of spatial memory impariment in DLD). In contrast, most studies examining visuo-perceptual memory tasks (requiring individuals to attend to and recall specific visual details, such as colour, shape, or object, and requiring ventral visual stream processing) report that DLD children perform worse than neurotypical peers, suggesting a deficit in visuo-perceptual memory (Bavin et al., 2005; Botting et al., 2013; Kleemans et al., 2012; Leclercq et al., 2012). Thus, when applying this neural network approach to the literature examining visual memory difficulties in DLD, the results become less heterogeneous and much more interpretable, and hence we have applied this perspective to interpreting the findings of the current review.

## The Current Review

The aims of this systematic review were to evaluate the literature examining the association between visual memory and vocabulary in neurotypical children (aged 2- to 12-years), and to explore in greater depth the potential impact of three important moderators: age, choice of visual memory task, and aspects of vocabulary measurement. Although a significant focus of the previous literature has been children with language difficulties, we chose to examine neurotypical children as current understanding of visual memory and vocabulary associations in this population is limited.

It was important to consider the possible impact of age, given the wide age range of our sample (2- to 12-years) that covers a period of extensive brain growth and development (Courchesne et al., 2000; Shaw et al., 2008), as well as the beginning of formal education. We chose to focus on this preschool and primary/elementary school period as the most exponential growth in vocabulary has been previously observed across these groups, with general vocabulary growth assessed via traditional, concrete tasks reported to plateau into the high school years (Duff et al., 2015; Ricketts et al., 2020). There is also a considerable literature demonstrating the substantial growth in visual memory and naming abilities over this period (Alloway & Alloway, 2013; Anderson & Lajoie, 1996; Buss et al., 2018; Gathercole et al., 2004; Pickering et al., 2001). On the other hand, some previous research suggests that the strength of memory-vocabulary associations decreases with age (Gathercole et al., 1992; Henry & Maclean, 2003; Rispens & Baker, 2012; Verhagen et al., 2019). Either way, this literature highlights the need to consider how age may impact the association between visual memory and vocabulary over the preschool and primary/elementary school years. Thus, we hypothesised that age would be a significant moderator, with findings likely weaker in older children compared to younger children, and assessed for this via meta-regression where possible.

Secondly, as discussed above, we grouped studies according to the aspect of visual memory being assessed (i.e., spatial or visuo-perceptual aspects, as they relate to dorsal and ventral visual stream processing; Goodale & Milner, 1992). We hypothesised, based on findings in DLD children, that visuo-perceptual memory tasks would be most closely related to vocabulary, with spatial tasks showing a weaker association.

Finally, as the association between visual memory and vocabulary may vary depending on the type of vocabulary assessed, and may be confounded by the frequent reliance on visual materials (e.g., pictures of objects or visual scenes) to assess vocabulary, we aimed to consider both vocabulary type (receptive or expressive) and modality of vocabulary assessment (visual or verbal) as potential moderators. We hypothesized that visual memory would be more strongly associated with receptive than with expressive vocabulary (based on a small number of previous findings, e.g., Nickisch & Von Kries, 2009), and that visually-based vocabulary tasks would be more strongly associated with visual memory than verbally-based vocabulary tasks.

## Method

This review was conducted and reported in accordance with the Preferred Reporting Items for Systematic Reviews and Meta-Analyses (PRISMA) guidelines (see supplemental document Table S1 for PRISMA checklist; Moher et al., 2009; Page et al., 2021). Prior to commencing the review, a protocol was pre-specified and registered with PROSPERO (registration number CRD42019125132; initial registration dated 26/06/2019; Pickering et al., 2019). A summary of amendments made to the protocol are presented in the supplemental document (Table S2). To be included in the systematic review, studies were required to report the correlation between visual memory and vocabulary in a sample of neurotypical children aged between 2- and 12-years. Specific details of eligibility criteria are provided below.

### Information Sources and Search Strategy

Studies were identified through searching the following electronic databases: PsychINFO (Ovid, 1806 to present), Medline (Ovid, 1946 to present), EMBASE (Ovid, 1947 to present), PubMed, Scopus (Elsevier), Web of Science (ISI), ERIC (ProQuest), and Cochrane Library. The search strategy included both keywords and MeSH terms as appropriate. The initial database search was run on the 17^th^ and 18^th^ of April 2019, with an updated search conducted on the 1^st^ of October 2020. In addition, hand searching was conducted from the reference lists of those studies that met inclusion criteria.

The search strategy was developed in consultation with an academic librarian and was designed to capture a broad array of studies that included measures of visual memory and vocabulary in a child sample (including studies where this was not the primary aim). An overview of the search strategy is presented below. The specific search strategy for all databases has been provided in the supplementary document (Table S3).

1. Visu* OR spati* OR “visu*spati*” OR “spati*visu*”
2. Memory OR “short term memory” OR “working memory” OR “complex memory” OR “declarative memory” OR “long term memory” OR STM OR WM OR LTM
3. 1 AND 2
4. Vocabulary OR lexicon OR “mental lexicon” OR “word knowledge” OR comprehension OR words
5. Child*
6. 3 AND 4 AND 5

### Eligibility Criteria and Study Selection

Eligible studies were required to be peer-reviewed publications written in English, with no limits on year of publication. Other forms of publications, including conference abstracts, dissertations, book chapters, study protocols, and government reports were therefore excluded. Although excluding such ‘gray literature’ from our search may increase the possibility of publication bias within the included studies, other methods were employed to decrease publication bias, including additional quality measures and statistical analyses. Studies were required to include a sample of neurotypical children, which included studies where the neurotypical children formed a control group (i.e., they were not the primary focus of the study). We initially aimed to include children from birth to 12-years, 11-months old [12;11], however, the scarcity of studies of children under 2-years prevented inclusion of this age group, and thus the final age range was 2;0-12;11. Studies that did not consider neurotypical children separately from clinical populations, or included participants 13-years and older (whether all or part of the total sample), were excluded. Additionally, given strong evidence that the language and memory abilities of bilingual children differ from monolingual children (see Grundy & Timmer, 2017, for a review), participants were required to be monolingual, with no restrictions on primary language spoken (as primary language was not expected to impact results). Studies were included if they employed both a measure of visual memory and an objective cognitive/behavioural measure of vocabulary (i.e., beyond parental questionnaires of vocabulary knowledge that were excluded). Longitudinal studies were included if the visual memory and vocabulary measures were completed at same timepoint, and, for consistency with single-timepoint studies, only data from the first study timepoint was included. Studies that included only a visual memory measure, only a vocabulary measure, or completed both measures but at different timepoints, were excluded.

Study eligibility was assessed independently by two authors (HP and JP) and one research assistant (LR) using the data management software Covidance (Veritas Health Innovation, 2019). The titles and abstracts of each identified record were screened independently by either HP and LR (initial search) or HP and JP (second search), to determine whether to reject the study or accept the study for further review. HP and JP then independently reviewed the method and results sections of each potentially relevant study to determine whether the study met the pre-specified inclusion/exclusion criteria. Disagreements were settled by consensus. The decision to reject a study was determined using a custom hierarchy of seven exclusion reasons:

1. Full-text document unable to be sourced,
2. Study not available in English,
3. Study design did not meet criteria (case-study, intervention study, longitudinal study with memory and vocabulary measures taken at different timepoints) or not a peer-reviewed publication (conference abstract, book chapter, thesis),
4. Population did not meet criteria (participants older than 12;11, no neurotypical group, bilingual or mixed sample),
5. Study did not include a measure of visual memory,
6. Study did not include an objective cognitive/behavioural measure of vocabulary,
7. Study did not report required correlation statistic (a bivariate correlation between, visual memory and vocabulary in a neurotypical sample).

### Quality Assessment

Quality assessment was completed at the study level using the Appraisal tool for Cross-Sectional Studies (AXIS tool; Downes et al., 2016). The AXIS tool assesses study quality with 20 items spanning five subsections: introduction, method, results, discussion, and miscellaneous (e.g., ethics). In order to compare quality across studies, outcomes were converted to a percentage, with a higher percentage representing a higher quality study. Percentages were calculated against the number of items applicable to the particular study (i.e., excluding items answered as “Not Applicable”). Ratings of 75% or higher represent a high study quality, values of 50–74% a moderate quality study, and scores below 50% a poor-quality study. Only studies with a quality score of 50% or above were included in the review. Two additional quality measures specific to the current review were rated: 1) did the aim/s of the study match the aims of the current review (included to assess possible publication bias), and 2) did the authors indicate why their particular visual memory task/s was chosen. These additional quality measures did not contribute to the overall quality percentage. These assessments (the AXIS tool and custom questions) were completed by the first author (HP), and 35% (*n* = 9) were randomly and independently reviewed by the second author (JP) for quality assurance. The initial overall interrater reliability was 81% (ranging 73–95% for individual studies), with disagreements regarding the rating of individual items then settled by consensus.

### Data Extraction and Synthesis

Data was extracted by the first author (HP). For each study, the following information was extracted: study details (authors, publication date, aims); participant information (sample size, age [M, SD, range], language spoken); measures (name, description, and procedure for visual memory and vocabulary measures); and outcomes (correlation coefficients, significance values). Where information was unclear or not available in the publications, attempts were made to contact the authors for additional information.

A task was considered to measure visual memory if the following conditions were met: 1) the study authors described the task as a measure of memory, and 2) the task description indicated that the content of information being processed and recalled was purely visual (i.e., visuo-perceptual details or spatial information) and did not additionally require processing or recall of non-visual information (i.e., auditory-verbal information). Vocabulary measures were defined as any language task that required participants to demonstrate their understanding of, or knowledge about, word meanings. Tasks were required to actively test participants knowledge (i.e., informant reports or checklists of known words were not included as a vocabulary task).

The primary outcome extracted was the bivariate correlation between a visual memory measure and a vocabulary measure. Where studies provided more than one correlation (i.e., from either multiple relevant participant groups or where there were multiple outcomes for visual memory and/or vocabulary measures), all correlation coefficients were extracted. Significance values were extracted where available for inclusion in relevant results tables. Whilst the inclusion of multiple outcomes from a single study means that not all outcomes are independent, which may overestimate the overall effect and underestimate error (Borenstein et al., 2009), sensitivity analyses were conducted, where appropriate, to overcome this (Brown & Tinsley, 2000).

To synthesise the data, the following categorisations were made by two authors (HP and SC) regarding the properties and procedures of the visual memory tasks:

1. Type of memory being measured (short-term memory, working memory, learning, or long-term memory),
2. Content of information to be remembered (spatial, visuo-perceptual, or both [visuospatial]),
3. Type of information processing (sequential/temporal or concurrent), and,
4. Response measure (sequential span, item recognition, item recall, change detection, or executive judgement).

Categorisations of each visual task used in the included studies were then independently reviewed by the second author (JP). Initial interrater reliability was 86%, with disagreements then settled by consensus. This information was then used to group visual memory tasks into categories for data analysis. Categorisations were made using the task description and procedure provided in the included studies, and by consultation with relevant task manuals. A summary of this information is presented in Table 1.

**Table 1:** Summary of Visual Memory Tasks and Categorisation for Data Synthesis

**Task Name; Citation**	**Task Description**	**Type of Memory**	**Content of Information**	**Type of Processing**	**Response Measure**	**In MA?**	**Citation/s in Review**
Block Recall (WMTB-C); Pickering and Gathercole (2001)	Participants are shown a board on which nine identical cubes (blocks) are fixed in a random arrangement. The experimenter taps out a sequence on the blocks, and the participant is required to tap the blocks back in the same sequence.	STM	Spatial	Sequential	Span	Y – TS	Critten et al. (2018)
Box Span Task; Michas and Henry (1994)	Participants are shown a set of nine boxes placed randomly on a sheet of paper. The experimenter points to sequences of boxes, and the participant is asked to point to the same boxes in the same sequence.	STM	Spatial	Sequential	Span	Y – TS	Michas and Henry (1994)
Colour Memory (focal and non-focal)^a^; Laws (2002)	Participants are shown a series of coloured tiles (1–8) for 5-seconds, and must then identify the tiles from a response set (containing distractors). One version uses focal colours (common chromatic colours, e.g., blue, green), and a second version uses non-focal colours (hues between common colours, e.g., turquoise, aqua).	STM	Visuo-Perceptual	Concurrent	Item Recognition	Y – CA (V)	Laws (2002)
Colour Recall; Schmid et al. (2008)	Participants are presented with a sequence of coloured discs on a computer screen and asked to recall the sequence in reverse order. Presentation time for each disc is one second.	WM	Visuo-Perceptual	Sequential	Span	Y – TS	Studer-Luethi et al. (2016)
Corsi Blocks; Corsi (1972)	Participants are presented with a series of nine randomly spaced blocks mounted on a wooden board. The experimenter taps a sequence of blocks, and the participant is required to reproduce the same tapped sequence. In a computerised adaptation (Cornu et al., 2018), boxes appear on a computer screen and change colour (from white to blue) for two seconds in a sequence. The participant responds by pointing to the boxes in the same sequence (Forward; STM), or in reverse sequence (Backward; WM).	Forward: STM	Spatial	Sequential	Span	Y – TS	Adams et al. (1999); Cornu et al. (2018); Laws (2002); Senese et al. (2020); van der Graaf et al. (2016)
		Backward: WM					
Corsi Blocks [adapted]; Xu and Lefevre (2016)	Between two and nine green circles are presented on an iPad screen. A sequence of circles is lit up for about 200ms each, and the participant is asked to reproduce the sequence by touching the locations in the same order.	STM	Spatial	Sequential	Span	Y – TS	Montoya et al. (2019)
Dot Locations (CMS); Cohen (1997)	Participants are presented with a 4x3 grid containing six blue dots for five seconds, and are asked to then replicate the array on a blank grid. This is repeated three times (with the same array each time). A distractor array of six red dots is then presented for five seconds (which they must recreate), following which they must again create the initial array of blue dots a final time. Scores are summed across all trials.	Multi-trial learning	Spatial	Concurrent	Item Recall	N	Malone et al. (2020)
Dot Matrix (AWMA); Alloway (2007)	Participants are shown a series of 4x4 matrices, containing between one and seven red dots, for two seconds. Then, they are shown a blank matrix and must recall the position/s of the dots.	STM	Spatial	Concurrent	Item Recall	Y – CA (S)	Blom et al. (2014)
Fish Visual Patterns Test (ViP); Stokes et al. (2017)	Participants are presented with 2-5 fish in fishbowls (up to 10 fishbowls) for five seconds. Then, they are shown the same number of empty fishbowls and asked to indicate where the fish had been located.	STM	Spatial	Concurrent	Item Recall	Y – CA (S)	Stokes et al. (2017)
Hand Position Imitation (NEPSY-II) [adapted]; Korkman et al. (2007)	Participants are presented with a progressively complex sequence of 18 hand/finger positions on a computer screen, and asked to imitate each one.	STM	Visuospatial	Sequential	Item Recall (visuo-motor)	N	Barbosa et al. (2017)
Location Memory; Bock et al. (2015)	Participants are presented with a square box divided into four regions. They watch the researcher place 20 objects on marked locations in the box; the objects are then removed and the participants is asked to place them back one at a time in random order. This is repeated until the child can correctly place all objects on the same trial. Then, the markers and boundaries are removed, and the child attempts to place all items a final time. Errors are used a score (measured as the distance between where each item was placed and where it should have been).	Multi-trial learning	Visuospatial	Sequential & Concurrent	Item Recall	N	Bock et al. (2015)
Memory for Designs (NEPSY-II); Korkman et al. (2007)	Participants are presented with a picture of a 4x4 grid containing a number of designs (4-8) and given 10-seconds to memorise the designs and their locations. Then, they are presented with a grid and cards (with one card for each correct design and one distractor card per design), and asked to recreate the original array. This is repeated over four trials, with new designs occasionally added to the array. Points are awarded for having the correct design on the grid (“content” score), having a design in the correct location (“spatial” score), and a “bonus” score for having the correct design in the correct location. Score are summed across the four trials (“total” score).	Multi-trial learning	Visuospatial	Concurrent	Item Recall & Recognition	N	Veraksa et al. (2018)
n-back (shapes); Gangopadhyay et al. (2016), Jaeggi et al. (2010)	Participants are shown a series of shapes on a computer screen and asked to indicate (with a button press) when a shape matches that shown one shape before (i.e., when the same shape appears twice in a row).	WM	Visuo-Perceptual	Sequential	Item Recognition & Executive Judgment	N	Yoo and Yim (2018)
Odd One Out (AWMA); Alloway (2007)	Participants are presented with three shapes in a row of boxes for two seconds. First, participants must identify the ‘odd-one-out’ shape. At the end of each trial, participants are presented with three empty boxes and asked in indicate, in sequence, the location of each odd-one-out shape. The number of items to be recalled (in sequence) increases progressively over successive blocks (with up to six trials per block).	WM	Visuospatial	Concurrent & Sequential	Executive Judgement & Item Recall	Y - EJ	Blom et al. (2014)
One-Shape Array Memory Task; Cowan et al. (2011)	Participants are shown a 3x4 gird where 2-6 coloured circles will appear for 500ms. After a 500ms break, the grid reappears with only 1 circle remaining, and the child must decide between: 1) no change (the circle is the same colour and in the same square as the original array), 2) spatial change (the colour appeared on the original array but has moved locations; the child must then indicate where it originally appeared), and, 3) colour change (a new colour that was not present in the original array).	STM	Visuospatial	Concurrent	Change Detection	Y – CA (VS)	Obeid and Brooks (2018)
Rey-Osterrieth Complex Figure Test (ROCFT); Rey and Osterrieth (1993)	Participants are initially presented with a complex visuospatial figure and asked to draw the figure on a separate page (whilst the figure remains in view). Then, after an immediate (2-3-minute, STM) and long (20-30-minute, LTM) delay, participants are asked to draw the figure again (without the figure in view).	Immediate: STM	Visuospatial	Concurrent	Item Recall	N	Batnini and Uno (2015)
		Long: LTM					
Spatial Working Memory (CANTAB); Cambridge Cognition (2006)	Participants are presented with a number of coloured boxes on a screen. They must ‘search’ the boxes (by selecting them) to find special tokens hidden in the boxes. Once a token is found under a box, a new token is placed under in a difference box, and participants much keep searching until a predetermined number of tokens are found. Once a token is found in one box, it will not be found there again within the same trial. Errors (re-searching previous boxes) are used as a score.	WM	Spatial	Concurrent	Executive Judgement & Item Recognition	N	Wilson et al. (2018)
Symbol Span (WMS-IV); Wechsler (2013)	Participants are presented with an array of symbols for 5-seconds. Then, they are shown a second array and are asked to identify the items from the first array in the same order (left-right).	STM	Visuospatial	Concurrent	Item Recognition & Span	Y	Palombo and Cuadro (2020)
Visual Matrix, Swanson Cognitive Processing Test; Swanson (1996)	Participants are presented with a series of dots in a matrix for 5-seconds. The matrix is then removed, and the participant is asked a ‘process question’ (e.g., how many dots in the first column?). After answering this question, the participant is asked to draw the dots in the correct boxes on a blank matrix. Matrices range from 4 squares and 2 dots to 45 squares and 12 dots.	WM	Spatial	Concurrent	Executive Judgement & Item Recall	Y - EJ	Vukovic and Lesaux (2013)
Visual Memory (TVPS-3(R)); Martin (2006)	Participants are shown 1 shape on a page for 5-seconds. On the next page, they have to identify that same shape from a set of 4 shapes.	STM	Visuo-Perceptual	Concurrent	Item Recognition	Y – CA (V)	Critten et al. (2018)
Visual Pattern Span; Wilson et al. (1987)	Participants are presented with a series of matrices in which half the cells are randomly filled. Participants are asked to recall the filled-in cells by recreating each design on a blank matrix. The number of filled cells to recall starts at two (on a 2x2) matrix and gradually increases.	STM	Spatial	Concurrent	Item Recall	Y – CA (S)	Adams et al. (1999)
Visual Sequential Memory (TVPS-3(R)); Martin (2006)	Participants are shown a specific shape (or set of shapes) on a page for 5-seconds. On the next page, they must identify the shape/set of shapes from 4 sets.	STM	Visuo-Perceptual	Concurrent	Item Recognition	Y – CA (V)	Critten et al. (2018)
Visual Reception (ITPA); Kirk and McCarthy (1961)	Participants are shown a stimulus picture for 5-seconds. The picture is removed, and participants must select a matching picture from a set of similar pictures.	STM	Visuo-Perceptual	Concurrent	Item Recognition	Y – CA (V)	Williams et al. (1977)
Visual Recognition Test (BAS); Elliott (1983)	Participants are shown a black-and-white drawing of one or more objects for 5-seconds. They are then asked to recognise those object/s from a new group (containing distractors).	STM	Visuo-Perceptual	Concurrent	Item Recognition	Y – CA (V)	Evans et al. (2008)
Visually Cued Recall; (Zelazo et al., 2002)	Participants are shown an array of 12 representational drawings, and a puppet indicates which of the picture/s it prefers. The array is hidden for 2-seconds, and then the participant must identify the preferred pictures.	STM	Visuo-Perceptual	Concurrent & Sequential	Item Recognition	Y – CA (V)	Séguin et al. (2009)
Working Memory – Lines; Seigneuric et al. (2000)	Participants are presented with a series of 3x3 matrices containing two coloured dots. First, participants are asked to point to the correct square to make a ‘winning’ line, whilst trying to remember the colour and position of the completed line. After each set of matrices has been presented, participants are presented with a blank matrix and the colours and asked to place (in sequence) all the lines.	WM	Visuospatial	Concurrent & Sequential	Executive Judgement & Item Recognition	Y - EJ	Seigneuric et al. (2000)
Working Memory Matrices – Sequential & Simultaneous; Lanfranchi et al. (2004)	Sequential: participants are presented with a 3x3 or 4x4 matrix, and the experimenter shows a frog making a path through the matrix (“jumping” between cells, one cell per second). Participants are then asked to reproduce the sequence in order	STM	Spatial	Sequential	Span	Y - TS	Meneghetti et al. (2020)
	Simultaneous: participants are shown either a 2x2 or 4x4 matrix, with some cells filled green, for 8-sections, and then asked to reproduce the green cells on a blank matrix.			Concurrent	Item Recall	Y – CA (S)	

*MA* meta-analysis, *WMTB-C* working memory test battery for children, *STM* short-term memory, *TS* temporal span, *CA* concurrent array, *V* visuo-perceptual, *WM* working memory, *CMS* children’s memory scale, *AWMA* automated working memory assessment, *S* spatial, *NEPSY-II* NEuroPSYchological assessment of the school-aged child, *EJ* executive judgement, *VS* visuospatial, *LTM* long-term memory, *CANTAB* Cambridge neuropsychological test automated battery, *WMS-IV* Wechsler memory scale, *TVPS-3(R)* test of visual perception skills, *ITPA* Illinois test of psycholinguistic abilities, *BAS* British ability scales

^a^Focal colours are common chromatic colours (e.g., blue, green), and non-focal colours are hues between common colours (e.g., turquoise, aqua)

Similarly, for vocabulary, the following judgements were made by two authors (HP and SC) regarding the properties and procedures of the vocabulary tasks:

1. Vocabulary type (receptive or expressive), and
2. Modality of information presented (visual or verbal).

A summary of this information is presented in the supplemental documentation (Table S4). Where possible, this information was used for subgroup analyses.

### Data Analysis

First, bivariate correlation coefficients from included studies were standardised to Fisher’s *z* to account for differing sample sizes and enable comparisons between studies. Standard errors were calculated using study sample sizes. Pooled correlation results were categorised as weak (.1–.3), moderate (.4–.6), or strong, (.7–.9) (Akoglu, 2018).

Studies were then grouped for analysis in two different ways. Initially, included studies were grouped according to vocabulary type (receptive or expressive), as this had been our original intention (as per the original protocol; Pickering et al., 2019). However, when extracting data from included studies it became apparent that most vocabulary tasks were visually based and most aimed to measure receptive vocabulary, which provided little variability and thus seemed unlikely to be driving the inconsistent results published to date. Therefore, to provide a more thorough and interpretable analysis of potential moderating factors, we instead grouped studies according to the type of visual memory task. This decision was made during the data extraction process and before any data analysis had been conducted. Where there were three or more correlation coefficients within a particular subgroup of visual memory measures, these were subjected to a meta-analysis. Other studies that did not meet this criterion are discussed in a narrative review (with results tabulated). Where possible, age and vocabulary type (receptive or expressive) were investigated as potential moderators. However, as this is only statistically viable when there are 10 or more datapoints within a given analysis (Deeks et al., 2020; Thompson & Higgins, 2002), such moderators could not be considered in most visual memory subgroups.

Statistical analyses were conducted using the JASP Meta-Analysis function (JASP Team, 2021). A random-effects model (restricted maximum likelihood, REML) was chosen *a priori*, as significant methodological variability between included studies was anticipated (Langan et al., 2019). The results are displayed in forest plots. Heterogeneity was primarily quantified using the *I*^*2*^ index, with values of 25%, 50%, and 75% considered to represent low, moderate, and high heterogeneity, respectively (Higgins & Thompson, 2002; Higgins et al., 2003). Additional measures of heterogeneity were also calculated and presented for transparency: τ^2^ (where a higher τ^2^ represents more heterogeneity) and the *Q* statistic (where the further *Q* is from the *df*, the higher the heterogeneity) (Borenstein et al., 2009). As some studies provided multiple datapoints within a single meta-analysis, sensitivity analyses were conducted where applicable, to ensure findings were not impacted by the non-independence of some data (Brown & Tinsley, 2000). As mentioned above, where possible (i.e., where there were 10 or more datapoints within a subgroup), meta-regression was used to investigate any heterogeneity (Deeks et al., 2020; Thompson & Higgins, 2002). To assess for possible publication bias, Egger’s regression test, funnel plots, and Rosenthal’s fail-safe N were presented (Egger et al., 1997; Rosenthal, 1979).

## Results

### Study Selection

The database searches returned 10,151 records, of which 5,339 were unique records (see Table S3 for a breakdown of records per database). Hand-searching the reference lists of included studies returned an additional seven references. Following independent title and abstract screening, 463 records were accepted for full-text review, while 4,876 were excluded as they clearly did not meet inclusion criteria. Full-text review excluded a further 424 records. A further 10 studies were initially considered to meet inclusion criteria, however, on closer inspection they were excluded due to key methodological differences that prevented comparison with other included studies. Of these 10 ‘near-miss’ studies: three reported only partial correlations (rather than bivariate correlations provided by the majority of included studies) (DeNigris & Brooks, 2018; Henry & Maclean, 2003; Rasmussen et al., 2009), two reported a composite memory score for analyses (where such composites comprised memory tasks that were judged to assess different aspects of visual memory, such as a spatio-temporal span task and a visuo-perceptual array task) (Lum et al., 2012; Vukovic et al., 2014), two reported age-standardised scores (rather than raw scores) (Alloway & Elsworth, 2012; Metcalfe & Stratford, 1986), one used a mixed vocabulary task (assessing both receptive and expressive vocabulary) (Alloway & Passolunghi, 2011), one combined receptive and expressive vocabulary scores for analyses (Joseph et al., 2005), and one reported z-scores (rather than raw scores) (Hooper et al., 2011). Characteristics and results of these 10 ‘near miss’ studies are provided in supplemental document (Table S5).

**Fig. 1:**
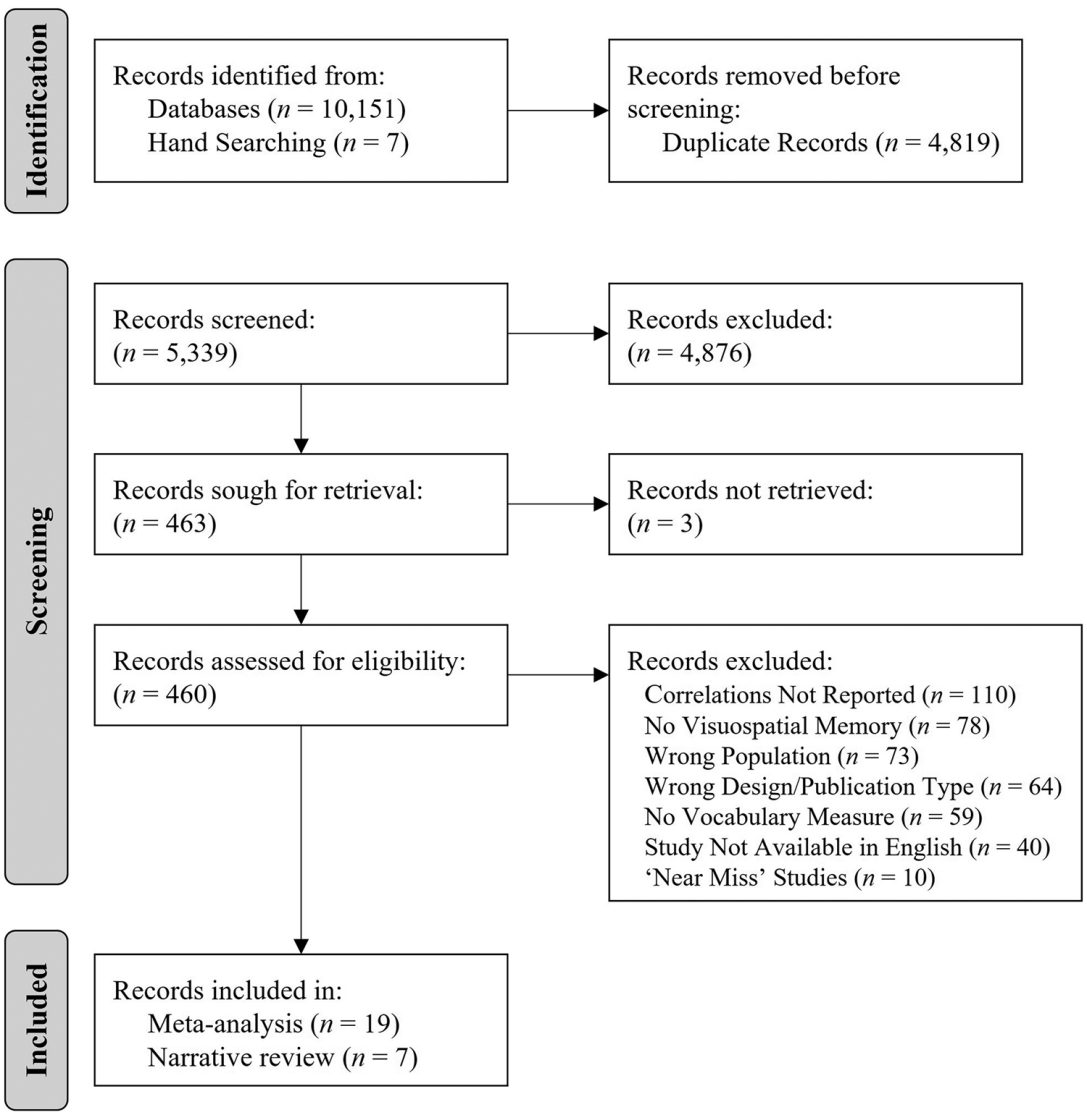
PRISMA flow diagram depicting how articles were selected for review

Thus, a total of 26 articles were identified for inclusion in the systematic review (see Fig. 1). The corresponding authors of five studies were contacted to provide further information for data extraction and synthesis; of these, four authors were contacted regarding participant information (Evans et al., 2008; Montoya et al., 2019; Palombo & Cuadro, 2020; Wilson et al., 2018), and one author was contacted for additional detail regarding their chosen vocabulary measure (Yoo & Yim, 2018). Three authors responded and provided the requested information (Evans et al., 2008; Montoya et al., 2019; Palombo & Cuadro, 2020) and no response was received from the other two authors (Wilson et al., 2018; Yoo & Yim, 2018). Information regarding the vocabulary measures from Yoo and Yim (2018) was ultimately able to be sourced from other papers using the same measure. Details of the author correspondence is provided in the supplemental document (Table S6).

### Quality Assessment and Risk of Bias

Quality assessment with the AXIS tool (Downes et al., 2016) was completed on the 26 included studies, and all studies were considered of sufficient quality to be included in the review and meta-analyses. When ratings were converted to percentages, scores ranged 60–88% (where a higher percentage represents a higher quality study; M = 74%). All studies provided adequate introductory sections and justified conclusions (items 1, 2, and 17), whereas no study included a sample size justification (item 2). A summary of the quality assessment is presented in Table 2, and a more detailed breakdown is provided in the supplemental document (Table S7).

**Table 2:**
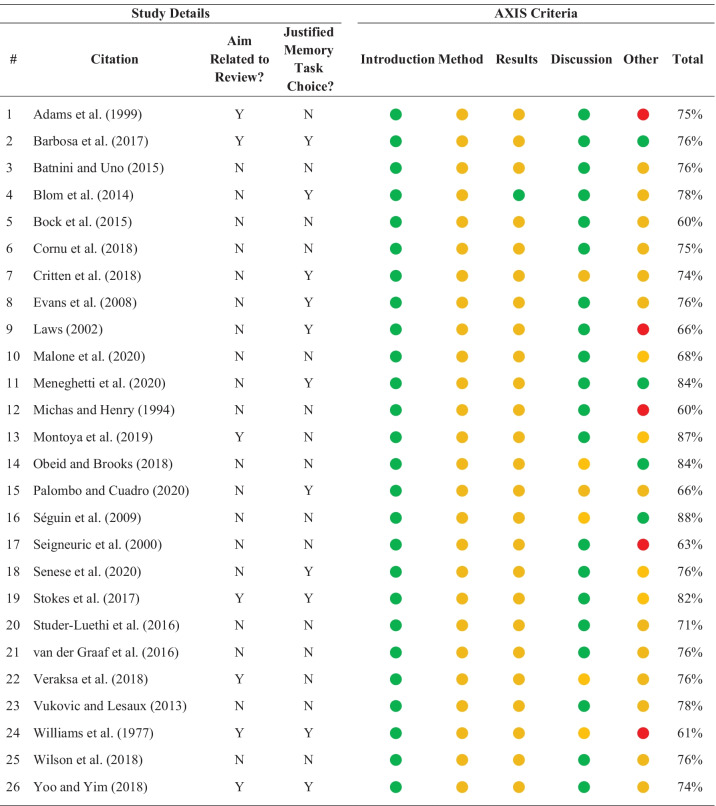
Risk of Bias for Included Studies

Green = all questions in that section answered yes. Orange = questions in that sections were answered with a mix of yes, no, and/or unclear. Red = all questions in that section answered no. See Supplemental Table S7 for details

*AXIS* appraisal tool for cross-sectional studies

In addition to the standardised quality assessment, two other quality measures were obtained (see Table 2). The first quality measure considered whether the aim of each included study matched the aim of the current review (i.e., examination of the association between visual memory and vocabulary and/or language abilities). Only seven of the 26 studies selected met this quality criteria which suggests a low risk of only including studies that have published significant results (i.e., publication bias), given that 19/26 studies published a result that was relevant to our review, but not the primary focus of the original study. Given our specific interest in the types of visual memory tasks used, the second additional quality measure considered whether the authors of the included studies justified their choice of a particular visual memory tasks (e.g., reliability, validity, known association with other variables of interest, previous research, or links to neuroscientific understanding of visual processing). Eleven studies provided a justification for their choice of visual memory task: three referenced previous research on a similar topic using the same visual memory task (Critten et al., 2018; Senese et al., 2020; Yoo & Yim, 2018), three referenced reliability and/or validity of their visual memory task in a relevant population (Barbosa et al., 2017; Evans et al., 2008; Palombo & Cuadro, 2020), two referenced manipulating aspects of their tasks, such as choosing colours that were easy or not easy to verbalise (Laws, 2002) or manipulating the memory load on a visual perception task (Williams et al., 1977), one referenced a known association with their outcome variable (Meneghetti et al., 2020), one designed a new task to be appropriate for their age-range (Stokes et al., 2017), and one referenced specifically assessing different aspects of memory (e.g., storage versus storage plus manipulation of information in working memory; Blom et al., 2014). No study provided a justification that related to neuroscientific knowledge of visual processing (e.g., why a spatial task was chosen over a visuo-perceptual task, or vice versa).

### Study Characteristics and Data Synthesis

Of the 26 included studies, 19 studies contributed 28 datapoints that were grouped into three meta-analyses (based on categorisation of the visual memory tasks, see below for details). The remaining seven studies utilised visual memory tasks that were unable to be meaningfully grouped together, and thus the 13 datapoints from these studies were considered in a narrative synthesis. Characteristics of the 19 meta-analysis studies are presented in Table 3, and characteristics and results of the remaining seven studies are presented in Table 4.

**Table 3:** Characteristics of Studies Included in Meta-Analyses

	**Study Details**		**Participants**			**Measures**	**Outcomes**	
**#**	**Citation**	**Language**	***N***	**Age M (SD); Range**	**Memory Task/s**	**Vocabulary Task/s**	**Meta-Analysis Category**	**Fishers’ *z* [95% CI]**
1	Adams et al. (1999)	English	66	5.05 (0.25); 4.5–5.5	1. Corsi Blocks	BPVT	1. Temporal Span	0.28 [0.03, 0.53]
					2. Visual Pattern Span		2. Concurrent Array (S)	0.19 [–0.06, 0.44]
2	Blom et al. (2014)^a^	Dutch	52	5.14 (0.18)	1. AWMA Dot Matrix	TT & TAK	1. Concurrent Array (S)	0.40 [0.12, 0.68]
					2. AWMA Odd One Out		2. Executive Judgement	0.30 [0.02, 0.58]
3	Cornu et al. (2018)	Luxembourgish	141	5.94 (0.44); 5–6	Corsi Blocks	Picture Naming Task	Temporal Span	0.21 [0.04, 0.38]
4	Critten et al. (2018)	English	32	8.5 (1.5); 6.17–11.58	1. WMTB-C Block Recall	BPVT	1. Temporal Span	0.50 [0.14, 0.86]
					2. TVP Visual Memory		2. Concurrent Array (V)	0.43 [0.07, 0.79]
					3. TVP Visual Sequential Memory		2. Concurrent Array (V)	0.58 [0.35, 0.81]
5	Evans et al. (2008)	English	76	4.44 (0.79); 3.0–6.08	BAS Visual Recognition Test	PPVT-III	Concurrent Array (V)	0.58 [0.35, 0.81]
6	Laws (2002)	English	16	4.54 (1.12); 2.17–6.92	1. Corsi Blocks	BPVT-II	1. Temporal Span	0.59 [0.05, 1.13]
					2. Colour Memory (Focal Colours)^b^		2. Concurrent Array (V)	0.91 [0.37, 1.45]
					3. Colour Memory (Non-Focal Colours)^b^		3. Concurrent Array (V)	0.33 [–0.21, 0.87]
7	Meneghetti et al. (2020)	Italian	30	5.49 (0.23); 5.17–6.00	1. WM Matrices - Sequential	PPVT-R	1. Temporal Span	0.26 [–0.12, 0.64]
					2. WM Matrices – Simultaneous		2. Concurrent Array (S)	.026 [–0.12, 0.64]
8	Michas and Henry (1994)	English	44	5.5; 5.17-6.17	Box Span Task	BPVS (short form)	Temporal Span	0.21 [–0.10, 0.52]
9	Montoya et al. (2019)	Spanish	419	4.4; 3.4–5.5	Corsi Blocks [adapted]	PPVT-R	Temporal Span	0.32 [0.22, 0.42]
10	Obeid and Brooks (2018)	English	63	8.17 (1.25); 6.0–10.67	One-Shape Array Memory Task	PPVT	Concurrent Array (VS)	0.41 [0.16, 0.66]
11	Palombo and Cuadro (2020)	Spanish	97	9.76 (0.26); 9–12	WMS-IV Symbol Span	WISC-IV Vocabulary	Concurrent Array (V)	0.19 [–0.01, 0.39]
12	Séguin et al. (2009)	English or French	1,693	3.38 (.05);	Visually-Cued Recall	PPVT-R	Concurrent Array (V)	0.39 [0.34, 0.44]
13	Seigneuric et al. (2000)	French	48	9.75; 8.67–10.58	Working Memory – Lines	Synonym Subtest of the California Test	Executive Judgement	0.76 [0.55, 0.97]
14	Senese et al. (2020)	Italian	142	8.8 (1.1); 7–11	Corsi Blocks (Forward)	WISC-IV Vocabulary	Temporal Span	0.20 [0.03, 0.37]
					Corsi Blocks (Backward)		Temporal Span	0.15 [–0.02, 0.32]
15	Stokes et al. (2017)	English	92	3.67 (.85); 2.0–5.25	Visual Patterns Test (ViP)	ROWPVT	Concurrent Array (S)	0.76 [0.55, 0.97]
						EOWPVT		0.81 [0.60, 1.02]
16	Studer-Luethi et al. (2016)	German	99	8.25 (.50);	Backward Colour Recall	Wortschataztest (Culture Fair Intelligence Test)	Temporal Span	0.02 [–0.18, 0.22]
17	van der Graaf et al. (2016)	Dutch	100	4.5; 4.0–5.17	Corsi Blocks	Verbal Meaning Test	Temporal Span	0.15 [–0.05, 0.35]
18	Vukovic and Lesaux (2013)^c^	English	75	6.83 (.41);	Visual Matrix (S-CPT)	WJ-III Picture Vocabulary	Executive Judgement	0.32 [0.09, 0.55]
19	Williams et al. (1977)	English	24	7.0; 5.16–8.91	ITPA Visual Reception Subtest	PPVT	Concurrent Array (V)	0.27 [–.016, 0.70]

*BPVS* British picture vocabulary test, *V* visuo-perpetual, *AWMA* automated working memory assessment, *TT* Toets Tweetaligheid (test for bilingualism), *TAK* Taaltoets Alle Kinderen (language test for all children), *S* spatial, *WMTB-C* working memory test battery for children;, *TVP* test of visual perception, *BAS* British ability scales, *PPVT* Peabody picture vocabulary test, *WM* working memory, *VS* visuospatial, *WMS-IV* Wechsler memory scale, *WISC-IV* Wechsler intelligence scale for children, *ROWPVT* receptive one-word picture vocabulary test, *EOWPVT* expressive one-word picture vocabulary test, *S-CPT* Swanson cognitive processing test, *WJ-III* Woodcock-Johnson III, *ITPA* Illinois test of psycholinguistic abilities

^a^Used Wave 2 data

^b^Focal colours are common chromatic colours (e.g., blue, green), and non-focal colours are hues between common colours (e.g., turquoise, aqua)

^c^Used Grade 1 data

**Table 4:** Characteristics and Results of Studies Included in Narrative Synthesis

**Study Details**			**Participants**		**Measures**		**Results**	
**#**	**Citation**	**Language**	***N***	**Age M (SD); Range**	**Memory Task/s**	**Vocabulary Task/s**	**Correlation*, p***	**Fishers’ *z* [95% CI]**
1	Barbosa et al. (2017)^a^	English	40	5.11 (0.54); 3.91–6.16	NEPSY-II Hand Position Imitation [adapted]	PPVT-III	*r* = .420, < .001	0.45 [0.12, 0.77]
		Mandarin	38	5.24 (0.66); 4.0–6.16			*r* = .216, *ns*	0.22 [-0.11, .055]
2	Batnini and Uno (2015)	Arabic	116	7.93 (0.43); 7–9	ROCFT Immediate Recall	ACTWA	*r* = –.046, *ns*	.05 [–0.14, 0.23]
					ROCFT Delayed Recall		*r* = –.008, *ns*	–0.01 [–0.19, 0.18]
3	Bock et al. (2015)	English	104	8.92 (1.42); 7–12	Location Memory	Vocabulary Definitions	*r* = .02^b^, *ns*	0.02 [–0.18, 0.22]
4	Malone et al. (2020)^c^	English	569	5.32 (0.36); 4.5–6.83	CMS Dot Locations	CELF-4 Expressive Vocabulary	*r* = .13, < .01	0.13 [0.05, 0.21]
5	Veraksa et al. (2018)	Russian	279	5.6; 5–6	NEPSY-II Memory for Designs	WPPSI Picture Naming	Content *r*_*s*_ = .110, *ns*	0.11 [–0.01, 0.23]
							Spatial *r*_*s*_ = .012, *ns*	0.01 [–0.11, 0.13]
							Bonus *r*_*s*_ = .029, *ns*	0.03 [–0.09, 0.15]
							Total *r*_*s*_ = .054, *ns*	0.05 [–0.06, 0.17]
6	Wilson et al. (2018)	English	126	5.42–12.92	CANTAB Spatial Working Memory	WASI Vocabulary	*r* = –.64^b^, < .01	–0.76 [–0.93, –0.58]
7	Yoo and Yim (2018)	Korean	20	8.23 (0.70); 7.0–9.5	n-back (1-back)	REVT	Receptive *r*_*s*_ = .20, *ns*	0.20 [–0.27, 0.68]
							Expressive *r*_*s*_ = .18, *ns*	0.18 [–0.29, 0.66]

*NEPSY-II* NEuroPSYchological assessment of the school-aged child, *PPVT* Peabody picture vocabulary test, *ROCFT* Rey-Osterrieth complex figure test, *ACTWA* Arabic comprehension test of abstract words, *CMS* children’s memory scale, *CELF* clinical evaluation of language fundamentals, *WPPSI* Wechsler preschool and primary scale of intelligence, *CANTAB* Cambridge neuropsychological test automated battery, *WASI* Weschler abbreviated scale of intelligence, *REVT* receptive & expressive vocabulary test

^a^Included two monolingual groups

^b^Memory score was an error score

^c^Used T1 data

#### Participant Characteristics

The 26 included studies involved a total of 4,545 participants, with 3,570 of these contributing to the meta-analysis. Participant ages ranged from 2;0 to 12;11 years old, although most studies (*n* = 16) focused on younger children (2–6 years). This may have been partially influenced by study inclusion criteria, as studies where the age range extended past 12;11 were excluded, even though in some of those studies the majority of children may have been under 12;11.

#### Visual Memory Task Characteristics

**Fig. 2:**
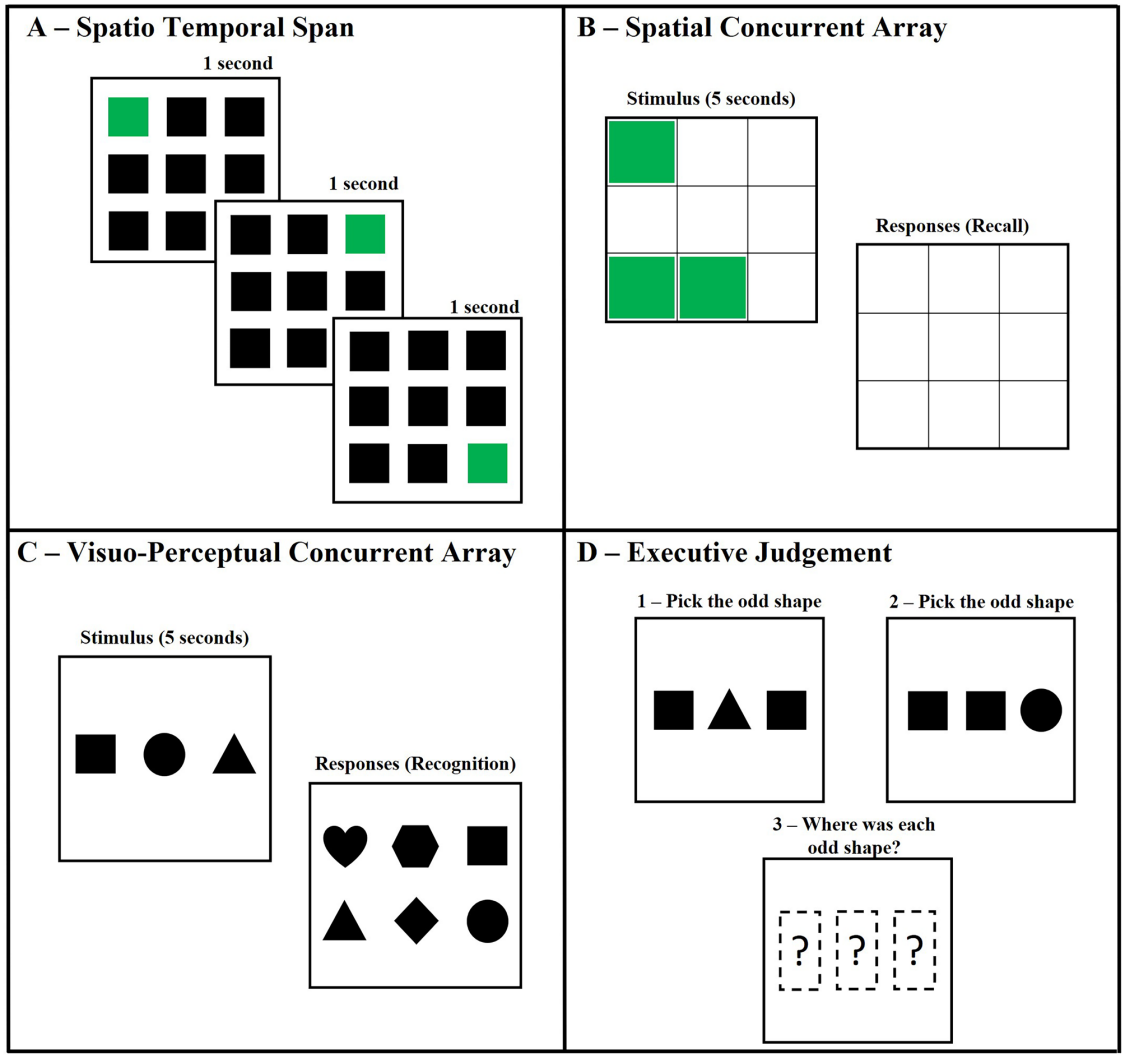
Example of each subtype of Visual Memory Task. *Note.* Panel **A**: Example spatio-temporal span task. Squares (green/grey) are highlighted one at a time (1 per second), and the participant must then re-create the temporal sequence. Panel **B**: Example spatial concurrent array task. A spatial display (left) is presented for a predetermined time (5-seconds), after which the participant must recall (recreate) the spatial display from a blank grid (right). Panel **C**: Example visuo-perceptual concurrent array task. An array of shapes (left) is presented for a predetermined time (5-seconds), after which the response array (right) is presented, and participants indicate which shapes they recognise from the original (first) array. Panel **D**: Example executive judgment task. Participants are first presented with a series of decisions to make (top row; pick which is the odd/different shape). After making all the decisions, they then must recall the location of each odd shape in the original order (i.e., triangle, then circle)

Measurement of visual memory was highly variable, with 27 different tasks used across the 26 included studies. Only one task, the Corsi block tapping task (Corsi, 1972), was used in more than one study. Based on the categorisation of the visual memory tasks (see Table 1 for details), four clear subgroups emerged: spatio-temporal span tasks, concurrent array tasks (which can be further separated into concurrent spatial arrays and concurrent visuo-perceptual arrays), and executive judgement tasks. These categorisations are described in detail below. Examples of each subtype of visual memory task are provided in Fig. 2.

Spatio-temporal span tasks (Fig. 2a) are distinct in that the information to be recalled is presented in a sequential-temporal fashion (i.e., one item at a time), with the participant asked to recall the position of the presented information in the correct temporal sequence. All temporal tasks included in the current review were spatio-temporal, in that the information to be recalled was spatial (i.e., the location of blocks on a table). The majority (8/10) of studies used the Corsi block-tapping task (or variant thereof; see Table 1 for details; Corsi, 1972), and 8/10 studies using this style of task required participants to recall the presented information in forward sequence (i.e., the order it was presented). Only two studies required participants to recall the information in reverse order. Because this instructional difference changes the theoretical interpretation of the task (as forward recall tasks are considered to measure short-term memory, whereas reverse recall tasks are considered to measure working memory; Lezak et al., 2012), results from these two studies are considered separately from the others.

Concurrent array tasks simultaneously present a visual array of information (i.e., all items at once) for a predetermined period of time (most commonly 5-seconds) and require either the spatial or visuo-perceptual details to be encoded. Following this, the participant is asked to either recall and reproduce the information, recognise the presented items from a second array (containing all the presented items and distractor items), or determine whether a second array differs from the first (change detection). Concurrent array tasks can be further categorised by the type of information to be remembered: spatial locations (Fig. 2b) or visuo-perceptual details (Fig. 2c).

Executive judgement tasks (Fig. 2d) involve executive decision making in addition to processing a concurrent array, often whilst also recalling the presented information in sequence. For example, judging a series of shapes to determine an ‘odd-one-out’, and after several trials recalling (in sequence) the location of each shape that had been ‘odd-one-out’ (Alloway, 2007). Thus, executive judgement tasks are considered more complex than temporal span and concurrent array tasks, given the multiple task demands to be processed and recalled.

#### Vocabulary Task Characteristics

Regarding included vocabulary measures, 14 studies measured receptive vocabulary, 10 studies measured expressive vocabulary, and two studies measured both. Seventeen different tasks were used across the 26 included studies. Nine different tasks measured receptive vocabulary, of which 7/9 were visually-based tasks (e.g., choosing a picture to match a spoken word), with one task (the Peabody Picture Vocabulary Test, PPVT; Dunn & Dunn, 2007) used in 7/16 studies. Similarly, of the eight tasks measuring expressive vocabulary, 6/8 were visually-based (e.g., picture naming), although the most common expressive vocabulary task (the Vocabulary subtest from the Wechsler intelligence scales; Wechsler, 2011a, b, used in 3/12 studies) was verbally-based (defining words). Further descriptions and categorisations of the tasks are provided in the supplemental document (Table S4).

### Results of Meta-Analyses

As per the pre-registered protocol (Pickering et al., 2019), studies were initially grouped according to vocabulary type (receptive or expressive). Following this, studies were then grouped according to the type of visual memory task (spatio-temporal span, concurrent array, executive judgement).

#### Receptive Vocabulary

For receptive vocabulary, results from 22 datapoints (15 studies, see Table 3) indicated a moderate, positive association with visual memory (pooled correlation = .36, 95% CI = .28–.45, *p* < .001, see supplemental document Fig. S1). However, there was significant, moderate heterogeneity (τ^2^ = .021, *Q*(21) = 47.46, *p* < .001, *I*^*2*^ = 66%). We attempted to account for this by considering age (mean age entered as a continuous variable) and vocabulary modality (visual or verbal) as potential moderators in a meta-regression, however, the model was not significant, and heterogeneity remained significant (*p* = .01, *I*^*2*^ = 56%). Full results of these analyses are presented in the supplemental document (Table S8).

#### Expressive Vocabulary

For expressive vocabulary, results from six datapoints (five studies, see Table 3) indicated a moderate, positive association with visual memory (pooled correlation = .31, 95% CI = .11–.51, *p* = .002). However, there was significant, very high heterogeneity (τ^2^ = .051, *Q*(5) = 29.78, *p* < .001, *I*^*2*^ = 84%). The small number of datapoints precluded using meta-regression to examine the heterogeneity, although inspection of the forest plot (see supplemental document Fig. S2) suggests that results from one particular study (Stokes et al., 2017) were very different from the others (influence measures indicated a high Cook’s Distance, 1.1, yet weighting was equivalent to the other datapoints), and may potentially be driving the heterogeneity.

Given these inconclusive and heterogeneous results, we chose to reconsider our grouping method, in order to better understand what may be driving the variability. This was achieved by grouping studies according to the type of visual memory task used. Studies were collapsed across vocabulary types, with vocabulary type (receptive or expressive) considered as a moderator where possible. These results are described below.

#### Spatio-Temporal Span Tasks

Of the 10 studies (contributing 11 datapoints, see Table 3) that used spatio-temporal span tasks, nine studies (contributing nine datapoints) were included in the meta-analysis, as they all used a forward variant of their respective tasks (indexing short-term memory). As only two studies (contributing two datapoints) used a backward variant of their span tasks (indexing working memory), their results were considered separately and, given the low number of studies, not pooled via meta-analysis.

Of the nine studies in the meta-analysis (forward variant of spatio-temporal span tasks), six studies used a physical block or box tapping task (Adams et al., 1999; Critten et al., 2018; Laws, 2002; Michas & Henry, 1994; Senese et al., 2020; van der Graaf et al., 2016), two studies used a comparable computerised/iPad tapping task (Cornu et al., 2018; Montoya et al., 2019), and one study (Meneghetti et al., 2020) showed a sequential pattern of squares in a matrix. Seven studies used a visually-based receptive vocabulary task, and the remaining two studies used expressive vocabulary tasks (one verbally-based and one visually-based). Additionally, the majority of studies (7/9) examined younger, mostly preschool age children (2–6 years), whereas the last two studies examined older children (6–11 years). Studies were of moderate to high quality (quality scores 60–87%, M = 75%).

**Fig. 3:**
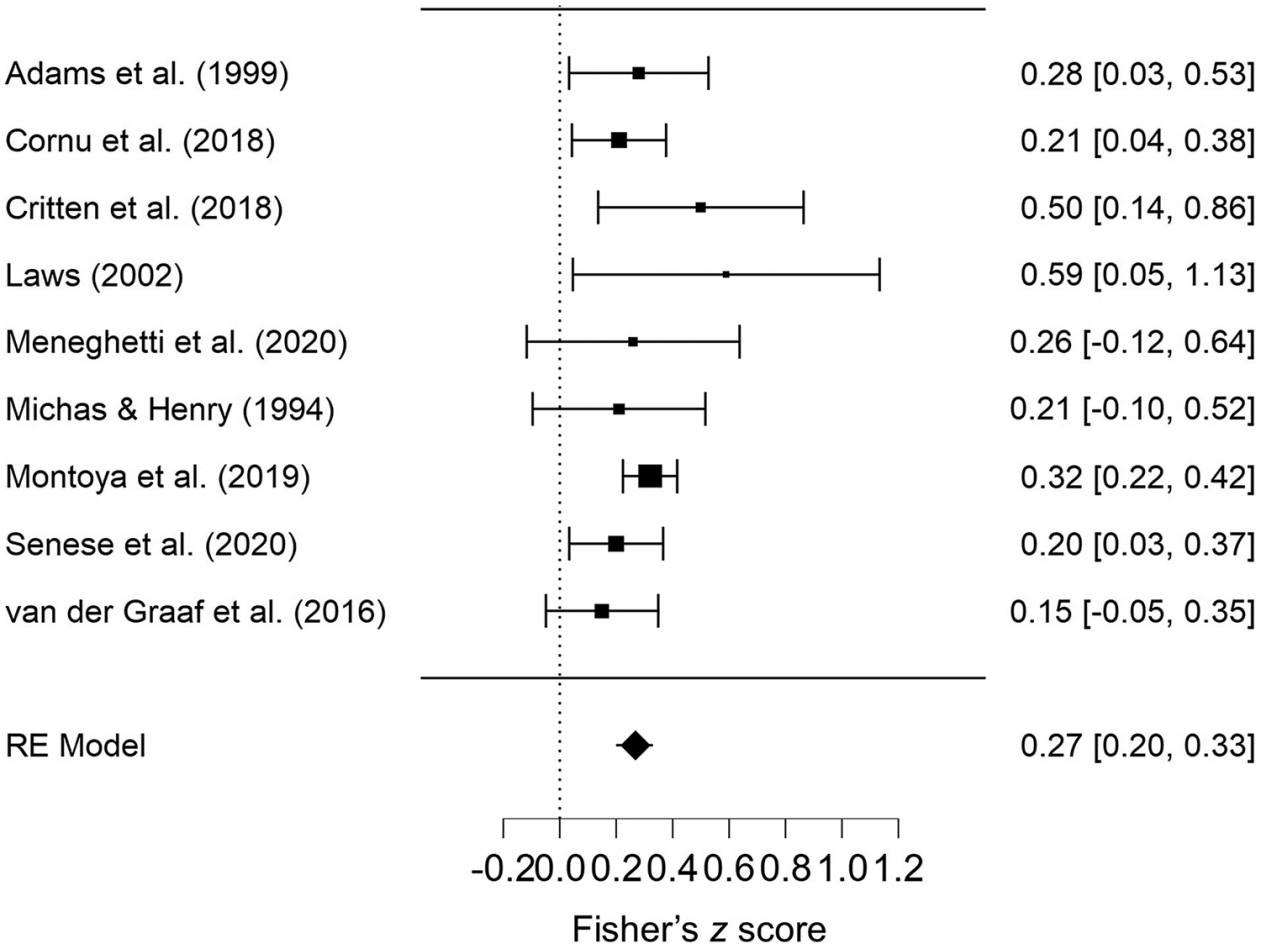
Forest Plot Showing Pooled Correlations (Fisher’s Z) for Spatio-Temporal Span Tasks. *Note.* RE = Random Effects

The results of the meta-analysis indicated a weak, positive relationship between spatio-temporal span memory tasks and vocabulary tasks (pooled correlation = .27, 95% CI = .20–.33, *p* < .001, see Fig. 3). Heterogeneity was low (τ^2^ = < .000, *Q*(8) = 6.64, *p* = .576, *I*^2^ = 3%).

Egger’s test indicated no publication bias (*p* = .536), and the funnel plot appeared symmetrical (see supplemental document Fig. S3a). Fail-safe *N* suggested 180 non-significant or unpublished studies would be required to nullify the significant result. One study (Montoya et al., 2019) was flagged as having a high influence on the results (Cook’s Distance = 1.19, weight = 40%), likely due to the large number of participants in this study (*n* = 419, next largest were Senese et al., 2020, *n* = 142, and Cornu et al., 2018, *n* = 141). However, when the analysis was re-run after removing this study (Montoya et al., 2019), there was little change in the result (pooled correlation = .23, 95% CI = .15–.32, *p* < .001).

The final two studies using spatio-temporal span tasks were not included in the above meta-analysis because they required participants to recall the spatio-temporal sequence in reverse order (rather than forward order, as was the case for all nine datapoints included in the meta-analysis). The first (Senese et al., 2020) used a backward block tapping task and a verbally-based expressive vocabulary task, whereas the second study (Studer-Luethi et al., 2016) used a backward colour recall task and a visually-based receptive vocabulary task. The studies were of moderate to high quality (71–75%). Participants in both studies were school-aged (average of 8-years-old). Both studies indicated a weak, non-significant association between backward spatio-temporal span tasks and vocabulary (Senese et al. Fisher’s *z* = .15 [–.02, .32], Studer-Luethi et al. Fisher’s *z r* = .02 [–.18, .22]).

#### Concurrent Array Tasks

Eleven studies contributed 14 datapoints (see Table 3) to an analysis of concurrent array tasks. All studies used a different task (see Table 1 for details). Four studies (Adams et al., 1999; Blom et al., 2014; Meneghetti et al., 2020; Stokes et al., 2017) with five datapoints examined spatially-based concurrent array tasks, where the participants were required to recall the spatial location of presented stimuli (e.g., which fishbowl contained fish). One study (Obeid & Brooks, 2018) required participants to recall both spatial and visuo-perceptual (colour) features of a presented array, and compare this to a second array (change detection). The remaining six studies (Critten et al., 2018; Evans et al., 2008; Laws, 2002; Palombo & Cuadro, 2020; Séguin et al., 2009; Williams et al., 1977) with eight datapoints examined visuo-perceptual concurrent array tasks, where the participant was required to recall perceptual details of the presented stimuli (e.g., different objects or colours). All eight tasks required participants to view an array with several perceptually different objects (or colours), and then recognise those items from a larger array (including distractors). One task (Critten et al., 2018, result b) additionally required participants to indicate the recognised series of objects in the order of presentation (e.g., which object was first in a presented line, which was second, etc). Nine of the eleven studies used a visually-based receptive vocabulary task, one study used a verbally-based expressive vocabulary task, and one study included both (visually-based) receptive and expressive tasks. The majority of studies (seven) examined younger, mostly preschool-aged children (2–6 years), with four studies examining predominately older children (5–11 years). Studies were of moderate to high quality (quality scores 66–88%, M = 77%).

**Fig. 4:**
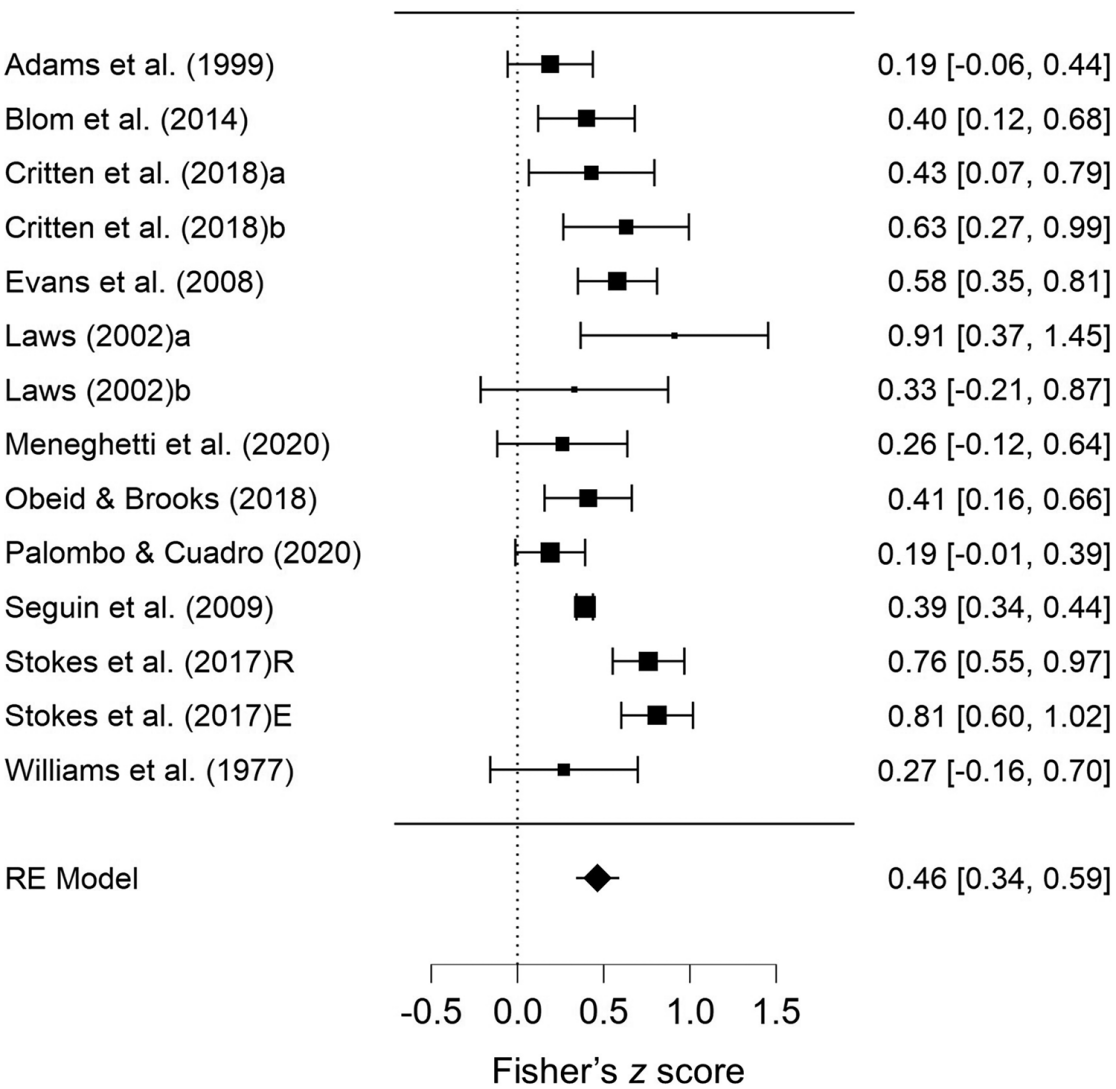
Forest Plot Showing Pooled Correlations (Fisher’s Z) for Concurrent Array Tasks (all). *Note.* RE = Random Effects

Initially, all eleven studies (14 datapoints) were examined in one meta-analysis, where the results indicated a moderate, positive relationship between concurrent array tasks and vocabulary tasks (pooled correlation = .46, 95% CI = .34–.58, *p* < .001, see Fig. 4). However, there was significant, moderate heterogeneity (τ^2^ = .032, *Q*(13) = 41.19, *p* < .001, *I*^*2*^ = 73%). Egger’s test indicated no publication bias (*p* = .875), and the funnel plot appeared symmetrical (see supplemental document Fig. S3b). No studies were overly influential. Fail-safe *N* was 1,225. As there were a number of non-independent datapoints within this analysis, a sensitivity analysis was conducted (Brown & Tinsley, 2000), which excluded (at random) the second datapoint for each applicable study and then re-ran the analysis. The result was very similar, suggesting little impact of non-independence: pooled correlation = .43, 95% CI = .30–.56, *p* = <.001; τ^2^ = .029, *Q*(10) = 28.72, *p* = .001, *I*^*2*^ = 72%.

Given the significant heterogeneity, additional analyses were undertaken. First, covariates were added into the initial model to determine if they could explain any of the heterogeneity. Both covariates – age (mean age of participants), and vocabulary type (receptive or expressive) – were non-significant (see supplemental document Table S9 for full results), and there was still significant, moderate heterogeneity (τ^2^ = .021, *Q*(10) = 22.37, *p* = .013, *I*^*2*^ = 54%). Therefore, subgroup analyses were conducted. Subgroup analyses considered the spatial and visuo-perceptual array tasks in two separate analyses (given these two types of information are processed in different brain networks; Goodale & Milner, 1992). One study (Obeid & Brooks, 2018) was not included in either subgroup analysis, as the visual memory task required recall of both spatial and visuo-perceptual information.

**Fig. 5:**
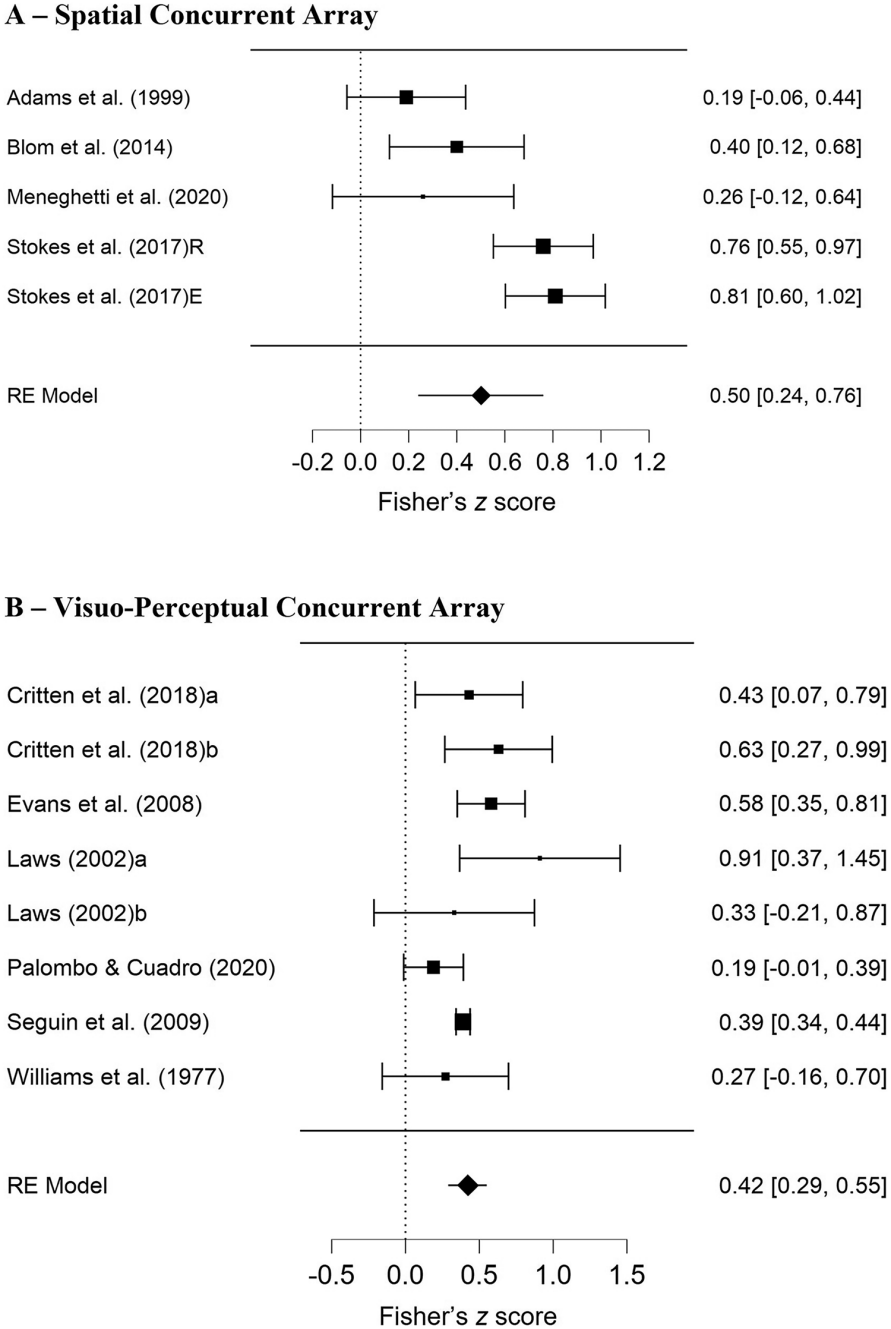
Forest Plots Showing Pooled Correlations (Fisher’s Z) for Concurrent Array Task Subgroups. *Note.* Random Effects. Panel **A**: Forest plot for spatial concurrent array tasks. Panel **B**: Forest plot for visuo-perceptual concurrent array tasks

For the spatial concurrent array tasks, results revealed a moderate, positive association with vocabulary tasks (pooled correlation = .50, 95% CI = .24–.76, *p* < .001, see Fig. 5a). Egger’s test indicated no publication bias (*p* = .097), and the funnel plot appeared symmetrical (see supplemental document Fig. S3c). Fail-safe *N* was 15. However, there was significant, high heterogeneity (τ^2^ = .068, *Q*(4) = .21.43, *p* <.001, *I*^*2*^ =80%). This was unlikely to be due to study quality, as all studies were of high quality (75–85%, M = 80%). Visual inspection of the forest plot (Fig. 4a) suggests that one particular study, Stokes et al. (2017), may be driving this variability (although weighting of those two datapoints was equivalent to the other studies). However, the small number of datapoints precluded analysing this statistically. Sensitivity analysis demonstrated a similar result: pooled correlation = .42, 95% CI = .15–.69, *p* = .002; τ^2^ = .055 *Q*(3) = 13.851, *p* = .003, *I*^*2*^ = 75%.

For visuo-perceptual concurrent array tasks, results revealed a moderate, positive association with vocabulary tasks (pooled correlation = .42, 95% CI = .29–.55, *p* < .001, see Fig. 5b). Heterogeneity was moderate, but non-significant (τ^2^ = .013, *Q*(7) = .11.95, *p* = .102, *I*^*2*^ = 49%). Egger’s test indicated no publication bias (*p* = .395), and the funnel plot appeared symmetrical (see supplemental document Fig. S3d). Fail-safe *N* was 426. Study quality was overall moderate (61–88%, M = 71%). Sensitivity analysis demonstrated a similar result: pooled correlation = .38, 95% CI = .26–.49, *p* = .<.001; τ^2^ = .008 *Q*(5) = 6.765, *p* = .239, *I*^*2*^ = 40%.

#### Executive Judgement Tasks

**Fig. 6:**
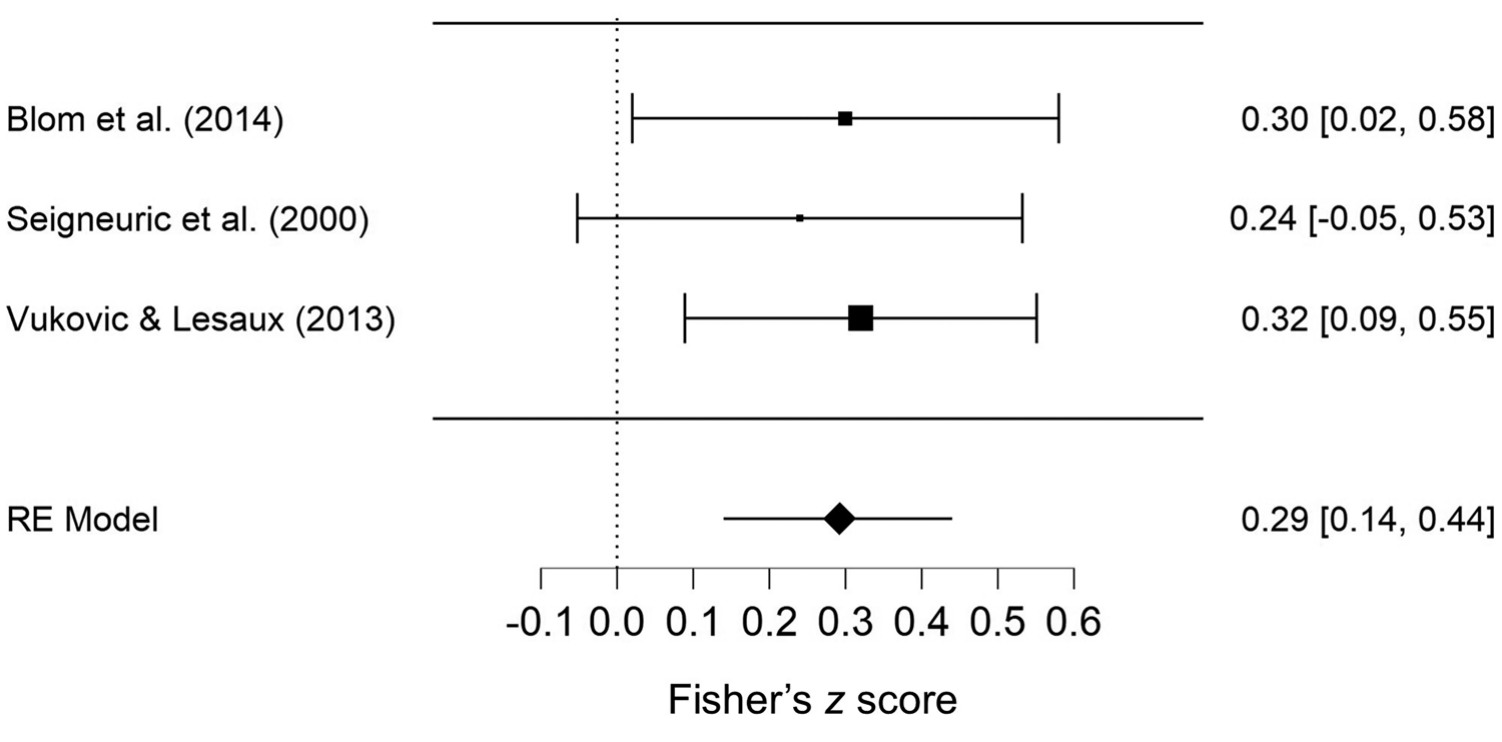
Forest Plot Showing Pooled Correlations (Fisher’s Z) for Executive Judgement Tasks. *Note.* RE = Random Effects

Three studies using executive judgement tasks were included in this analysis (Blom et al., 2014; Seigneuric et al., 2000; Vukovic & Lesaux, 2013). All studies used different executive judgment tasks and different types of vocabulary tasks (including a visually-based receptive vocabulary task, a visually-based expressive vocabulary task, and a verbally-based expressive vocabulary task; see Table 3 for details). Two studies recruited younger participants (5–6 years), while the third study had older participants (8–10 years). The results of the meta-analysis indicated a weak, positive association between executive judgement tasks and vocabulary tasks (pooled correlation = .29, 95% CI = .14–.45, *p* < .001, see Fig. 6). Heterogeneity was very low (τ^2^ = .000, Q(2) = .181, *p* = .912, *I*^*2*^ = 0%). Egger’s test indicated no publication bias (*p* = .738), and the funnel plot appeared symmetrical (see supplemental document Fig. S3e). No studies were flagged as being overly influential. Fail-safe N was 13. Study quality was moderate to high (63–87%, M = 73%).

### Results of Narrative Synthesis

Seven additional studies were eligible for inclusion in the current systematic review, however the tasks employed did not map onto the three categories described above, and differences between these seven studies precluded analysing them as a singular subgroup. Thus, they were not subjected to a meta-analysis, and are instead briefly summarised below. Study characteristics and results of these seven studies are presented in Table 4. Studies were of moderate to high quality (60–76%, M = 72%).

Three studies (Bock et al., 2015; Malone et al., 2020; Veraksa et al., 2018) used tasks that involved multi-trial learning of visuospatial information. As one task (Location Memory, Bock et al., 2015) used an error score as the outcome, results across the three studies cannot be directly compared. Correlations with expressive vocabulary tasks (verbally-based in Bock et al. and Malone et al., and visually-based in Veraksa et al.) overall showed no association. Two studies (Barbosa et al., 2017; Batnini & Uno, 2015) used visual memory tasks with a high load on visuo-motor skills (imitating hand movements and figure drawing), yet the results were variable and inconclusive. Wilson et al. (2018) used a complex visual search task, requiring spatial working memory and executive judgements. Although this shares some similarities with the category of executive judgment tasks, there was no requirement to recall the sequential presentation of the information, and the use of an error score again precluded comparison with other results. Better performance (less errors) was moderately correlated with expressive vocabulary. Finally, Yoo and Yim (2018) was the only study to use an n-back task, which requires continuous updating of task information in working memory. There were moderate, nonsignificant correlations with both receptive and expressive vocabulary.

## Discussion

This systematic review and meta-analysis aimed to assess the literature examining the association between visual memory and vocabulary in neurotypical children aged 2- to 12-years. Although strong evidence from infant studies shows that early visual attention and memory processes support later language and vocabulary abilities (Cetincelik et al., 2021; Mundy et al., 2003; Ortiz-Mantilla et al., 2008), research in older children has been highly variable to date (e.g., Vugs et al., 2013). Hence, we aimed to consider and elucidate this variability in three ways, by considering age, aspects of visual memory tasks, and aspects of vocabulary tasks. A total of 26 studies of moderate to high quality met the inclusion criteria, and 19 studies ultimately contributed to three separate meta-analyses.

### Developmental Factors

Age was an important factor to consider, given the large age range included in our meta-analysis (2- to 12-years). During this period, there is rapid and extensive brain growth and change (Courchesne et al., 2000; Shaw et al., 2008), as well as development of visual memory (Alloway & Alloway, 2013; Anderson & Lajoie, 1996; Buss et al., 2018; Gathercole et al., 2004; Pickering et al., 2001) and general vocabulary knowledge (Ricketts et al., 2020; Trautwein & Schroeder, 2017). Therefore, mean age was included as a continuous variable in our meta-regressions (although see below for limitations of this method). However, as meta-regression can only be conducted when more than 10 datapoints are available (Deeks et al., 2020; Thompson & Higgins, 2002), it was not always possible to consider age with statistical methods. In the two analyses where age *was* included as a moderator (our initial grouping where all receptive vocabulary tasks were considered together, and the overall concurrent array analysis), the contribution of age was not significant. One additional analysis of spatial concurrent array tasks showed a potential age effect upon visual inspection of the forest plot, where the results from one study of younger children (Stokes et al., 2017, mean age 3;8) appeared to show a much stronger correlation between spatial-array tasks and vocabulary compared to the three other studies of slightly older children (Adams et al., 1999; Blom et al., 2014; Meneghetti et al., 2020; mean ages 5;0–5;6). While the small number of studies in this analysis subgroup precluded statistical analysis of potential age effects, it does suggest that age-related effects likely occur, but were undetectable using current analysis methods. Indeed, such a finding – that the association between visual memory and vocabulary decreases with age – would be consistent with one previous study (Henry & Maclean, 2003), and with several other studies that have shown the association between auditory-verbal memory and vocabulary to decrease with age (Gathercole et al., 1992; Rispens & Baker, 2012; Verhagen et al., 2019). However, the exact impact of developmental factors requires further research with more appropriate study designs (e.g., longitudinal studies).

### Important Aspects of Visual Memory Tasks

To understand the potential impact of memory task selection on the association between visual memory and vocabulary, we grouped studies according to neuroscientific evidence that incoming visuo-perceptual information is primarily processed by the ventral “vision for perception” pathway, whereas spatial and temporal information is predominantly processed by the dorsal “vision for action” pathway (Goodale & Milner, 1992; Kravitz et al., 2011, 2013). This distinction is supported by both neuroimaging and behavioural studies of visual memory in children and adults (D'Esposito, 2007; Eriksson et al., 2015; Pickering et al., 2001; Simmering et al., 2015; Wager & Smith, 2003), and was also apparent within our results. For instance, spatio-temporal span tasks, which require sequential spatial processing likely supported by the dorsal visual stream, showed a weak association with vocabulary. In addition, spatial concurrent array tasks, where spatial distribution is expected to be preferentially processed by the dorsal visual stream, were variable (likely due to age factors discussed above). Further, consistent with our hypothesis, visuo-perceptual array tasks, theoretically associated with ventral visual stream processing, showed a moderate association with vocabulary. This is in line with neuroscientific understanding of both visuo-perceptual processing and semantics (word meanings), as both abilities are preferentially processed by the temporal cortex (Deldar et al., 2021). Notably, our findings highlight that the ability to perceive, process, and maintain in mind visuo-perceptual details of a given visual scene may support mapping the word to its meaning. Additionally, our findings are consistent with recent models of early word learning in toddlers, which highlight that key visual processes, such as joint visual attention (i.e., following another’s gaze), spatial attention (i.e., locating something or someone within a visual scene), visual and spatial working memory (i.e., maintaining aspects of the visual scene in mind), and multisensory association (i.e., mapping a spoken word to a specific object within a visual scene) support word-object mappings and vocabulary development in the first years of life (Cetincelik et al., 2021; Choudhury et al., 2007; Samuelson, 2021). Our results, whilst only assessing some of these aspects, highlight that visual processes continue to be associated with vocabulary development in older children, although our findings do suggest a stronger role for visuo-perceptual processes, rather than the spatial processes more commonly investigated and implicated in younger children. Indeed, as our findings did differ across the different types of visual memory tasks, and particularly between spatial and visuo-perceptual processing, they demonstrate that this distinction can affect research findings and that future research needs to consider these fundamental visual processing networks when assessing any visual cognitive ability.

Further, application of this neuroanatomical view (i.e., comparing spatial processing via the dorsal visual stream to visuo-perceptual processing via the ventral visual stream) facilitates understanding of the apparent variability in the existing literature examining visual memory abilities in children with Developmental Language Disorder (DLD). For instance, one of the most consistent findings is that DLD children perform comparatively to (and, in some cases, above) their age-matched neurotypical peers on spatio-temporal span tasks, such as Corsi-style block tapping tasks (Archibald & Gathercole, 2006b, 2007; Arslan et al., 2020; Botting et al., 2013; Briscoe & Rankin, 2009; Hutchinson et al., 2012; Lukács et al., 2016; Lum et al., 2012; Petruccelli et al., 2012; Williams et al., 2000; although see Bavin et al., 2005, and Jackson et al., 2020, for contrary evidence). Results regarding spatial array tasks are less clear, with some studies finding DLD children are impaired on such tasks (recalling the locations of sharks presented on a grid) relative to age-matched neurotypical peers (a deficit which appears to widen over time; Hick et al., 2005a, b), yet others finding that DLD children perform comparatively to their peers on two different spatial array tasks (recalling locations of dots in a grid, and recalling paths through a maze; Hutchinson et al., 2012; Lum et al., 2012; Vugs et al., 2017). This variability in past DLD research is also in line with our spatial array results. Regarding visuo-perceptual tasks, the evidence base is smaller but consistent in finding that DLD children are impaired (relative to age- and/or language-matched neurotypical controls) on tasks of abstract pattern recognition, pattern recall, and visual detail recall (particularly simple visuo-perceptual details) (Bavin et al., 2005; Botting et al., 2013; Kleemans et al., 2012; Leclercq et al., 2012). This result appears consistent with our finding that visuo-perceptual tasks are most clearly related to vocabulary abilities. However, it is important to note that children with DLD are a heterogenous group, and research criteria for inclusion into studies of DLD can vary significantly (Bishop et al., 2017; Reilly et al., 2014); thus, there may be other unknown factors contributing to how visual memory and vocabulary relate in this population. Further research comparing different types of visual memory tasks, in both DLD and neurotypical children, combined with careful consideration of the inherent differences in how visual information is processed in the brain, will be important steps to improve our understanding of how such visual memory processes may support vocabulary development.

Several other kinds of visual memory tasks were considered in the current review, including executive judgment tasks, multi-trial learning, visuo-motor memory tasks, and dynamic updating tasks (e.g., n-back tasks). Broadly speaking, none of these areas showed any clear association with vocabulary abilities, although this is based on a small number of studies (*n* = 1–3 per subtype). More research is needed in these areas to better understand potential relations with visual memory, in both neurotypical and DLD children.

### Consideration of Vocabulary Tasks

In addition to examining types of visual memory tasks, it is important to consider common aspects of vocabulary assessment. Of most relevance to the current review is the fact that most standardised measures of vocabulary (receptive or expressive) are visually based. This was the case for 13/17 vocabulary tasks considered in the current review. Using visual information to assist in the assessment of vocabulary and verbal language skills inherently implies that the child has the ability to process and understand the presented visual information in order to demonstrate their vocabulary knowledge. For instance, in most visually-based receptive vocabulary tasks, the child hears a spoken word and must match it to one of three or four pictures. Thus, they must understand the semantic meaning of the verbally-presented word, process the details of each picture, make a semantic decision about what each picture represents, and then compare this back to their understanding of what the spoken word means. This relies heavily on visuo-perceptual processing, which may have contributed to our primary finding (that visuo-perceptual memory tasks were moderately associated with vocabulary). Similarly, visually supported expressive vocabulary tasks, which typically ask the child to name or describe a picture or visual scene, require the child to be able to accurately process the picture/scene, access the name of the objects, and use verbally-based language to explain their understanding; in this instance, accurate visuo-perceptual and semantic processing must be intact in order for the child to provide an accurate verbal description. The ability to hold the visual information in mind, and access long-term semantic knowledge relating to that information (i.e., visual short-term, working, and long-term memory) would be required to enable successful completion of such tasks. By considering vocabulary assessment tasks in this wholistic way (i.e., the interaction and integration of visual and language demands of the tasks), we can better understand the various cognitive and developmental processes that underlie the target skills (i.e., understanding and using vocabulary appropriately), and ultimately better support individuals with weaknesses in these areas. However, more research using non-visual vocabulary tasks (see Table S4 in the supplemental document for examples) is required to fully elucidate the associations between visual memory and vocabulary without the confound of visual processing in vocabulary

Moreover, the aspects of vocabulary under examination are also important. Evidence indicates that visually based abilities, such as visuo-perceptual short-term memory and broader visual intelligence, have been specifically associated with receptive language impairment and not expressive language impairment (Nickisch & Von Kries, 2009; Saar et al., 2018), suggesting there may be differences between how visual memory relates to the different types of vocabulary. However, given few included studies considered expressive vocabulary, and the confound of visually based assessment discussed above, further research is needed in this area to clarify the relationships.

### Limitations and Future Directions

This review has several limitations that need to be considered. Firstly, the results analysed were correlational. We chose to use correlational data as our primary interest was the association between visual memory and vocabulary, but this choice means that more research is needed to determine the exact nature of the relationship between visual memory (or types thereof) and vocabulary. Further, only bivariate correlations were used; whilst this was necessary to ensure consistency across the statistics being compared, it does not control for potential confounds, such as intellectual abilities, or age. Whilst we attempted to account for age-related factors, this is difficult in meta-analyses where the included studies often each have a wide age range (as was the case for most studies included in our review). Of the two options available, using the mean age of participants as a continuous variable or creating a dichotomous variable of ‘younger’ and ‘older’ participants, we chose the former as the literature does not suggest a clear cut-point for grouping participants. It is important to acknowledge, however, that mean age may not be wholly representative of the sample in each study, and may not fully account for age-related variance, which may contribute to why significant age effects were not found. Further research better designed to answer age and developmental questions (such as cohort and longitudinal studies) would therefore be useful in advancing our understanding of the developmental course of visual memory and vocabulary relations.

From a statistical standpoint, it is also important to acknowledge that the relatively small number of studies meeting our inclusion criteria did not allow for the application of any quantitative statistical methods to compare our correlation coefficients and meta-analysis outcomes. This is because sample sizes within each visual memory subgroup were not sufficiently powered to conduct such statistical comparisons (Borenstein et al., 2009). Thus, we were instead restricted to more qualitative comparisons. Whilst statistical comparisons may have aided our understanding of the outcomes, they would also need to be interpreted in the context of heterogeneity, which was moderate to high in many of our outcomes; thus, statistical comparisons are unlikely have offered any additional, meaningful insight into our results at this time. Hopefully, future research comparing different aspects of visual memory will be able to include such comparisons.

This review is also limited by the kinds of visual memory tasks available in the current literature. This limitation in the studies available restricted both the power of analyses (as some subtypes of visual memory tasks contained only a few studies) and may also constrain theoretical interpretations in some instances. For example, the majority of tasks used in the literature are traditional working memory span tasks, predominantly based on the prominent multicomponent view of working memory (Baddeley, 2012; Baddeley & Hitch, 1974). However, more contemporary views of working memory are rejecting span and capacity-based models in favour of more attention-driven models that highlight the importance of flexibility and executive control, and incorporate neuroscientific understanding of dynamic working memory processes in the brain (e.g., Adams et al., 2018; Burgoyne et al., 2019; D'Esposito & Postle, 2015; Engle, 2018). Thus, moving forward, it will be important for research considering visual working memory and vocabulary to use tasks that best represent current theoretical understanding of relevant memory constructs and the neural networks that underpin their function.

Finally, as discussed above, future research into vocabulary (and broader language) development, and associations with visual memory, needs to carefully consider how particular vocabulary and memory tasks are chosen, as this is likely to impact results. Whilst our primary consideration of visual memory tasks was differentiating aspects of visual processing (e.g., spatial versus visuo-perceptual), more work considering how this may interact with other aspects of memory processing (e.g., working versus long-term memory) may be useful. It would also be useful for future research to examine whether the association between visual memory and vocabulary may vary depending on the language spoken. Whilst our included studies covered a range of languages (see Tables 3 and 4), and results appear consistent across the various languages, small sizes per language (other than English) precluded examining this as another moderator, and thus the potential impact is unknown. Additionally, the current review focussed on vocabulary development, and primarily development of general, concrete vocabulary (e.g., nouns and verbs which are easily depicted pictorially). This constitutes just one aspect of vocabulary, which is only one aspect of verbal language. We chose to focus on this type of vocabulary given it is the most commonly assessed (and used) in childhood, and because most of the previous work examining how memory (visual or auditory-verbal) may support language development has focused on this type of vocabulary. Future research should attempt to extend understanding of how visual memory (and visual cognition) supports language and vocabulary abilities more generally. Further, applying findings from the current review to children with DLD specifically would be beneficial.

### Conclusions and Implications

In summary, the current systematic review and meta-analysis sought to better understand the association between visual memory abilities and vocabulary in neurotypical children (up to 12 years). Meta-analyses revealed that visuo-perceptual array memory tasks were moderately associated with vocabulary, suggesting that children with a greater ability to process, hold in mind, and recall specific details of a presented visual scene (such as specific colours or objects) also have greater vocabulary knowledge (receptive vocabulary). In contrast, spatio-temporal and executive judgement tasks showed only a weak association with vocabulary. Other aspects of visual memory, including spatial concurrent array tasks, visuomotor memory tasks, multi-trial visuospatial learning, and dynamic visual memory, were inconclusive and require more research. From the available literature, other factors that may moderate this relationship, such as age, intellectual functioning, schooling, or variation in vocabulary task, also require further investigation.

From a clinical practice perspective, these results highlight the need to assess visual cognition in children presenting with language difficulties, as they may have co-occurring difficulties in this area (both visuospatial memory impairment as identified in the current review, and potentially reduced non-verbal intellectual functioning, see Gallinat & Spaulding, 2014). Such assessments would typically fall to neuropsychologists, who routinely assess non-verbal intelligence and visual memory (Lezak et al., 2012). Indeed, this would be in line with recent calls to consider DLD and language impairments from a broader neuropsychological perspective (rather than a narrow focus on speech/language deficits; Tomas & Vissers, 2019), although consideration of visual memory and broader visual cognition remains notably absent from such accounts. Further, given that interventions and supports for vocabulary development are often visually-based (Steele & Mills, 2011), understanding a child’s visual cognition is vital to ensuring these supports are appropriately implemented.

From a research perspective, this review also highlights the importance of understanding the different ways that visual memory can be measured, and the need for researchers to carefully consider their choice of visual memory task. In the current review, the only task included in more than one study was the Corsi block tapping task (Corsi, 1972), yet this type of task had only a weak association with vocabulary. Task choice, therefore, may have a considerable impact on the results, and the theoretical and clinical implications that stem from such findings. This is equally important for considering how vocabulary (and broader language abilities) are assessed. Thus, future research into both children with language impairment and vocabulary development more broadly should pay close attention to the various ways that visual information can be presented and encoded into memory, while ensuring that task selection accurately reflects the aspects of the visual memory system most related to the research question at hand. Ultimately, this will allow for a more detailed understanding of how memory abilities may support vocabulary development, and guide successful, targeted, and wholistic interventions for children with vocabulary and verbal language difficulties.

### Supplementary Information

Below is the link to the electronic supplementary material.

Supplementary file1 (PDF 1148 KB)

## Data Availability

Data included in the meta-analyses are available here: https://doi.org/10.26181/6125e5b438a49

## Code Availability

N/A
