# Supplementary material for: A Role for Visual Memory in Vocabulary Development: A Systematic Review and Meta-Analysis
Source: Neuropsychol Rev. 2022 Sep 22;33(4):803–33. doi: 10.1007/s11065-022-09561-4 (PMC10770228; doi:10.1007/s11065-022-09561-4)
Supplement: Supplementary file 1 — Supplementary file1 (PDF 1148 KB) [file 11065_2022_9561_MOESM1_ESM.pdf]

Supplemental Document

A Role for Visual Memory in Vocabulary Development: A Systematic Review and Meta-Analysis

Hayley E. Pickering, Jessica L. Peters, & Sheila G. Crewther

Table of Contents

Table S1: PRISMA Checklist 2

Table S2: Summary of Amendments to PROSPERO Protocol 5

Table S3: Full Search Strategy for all Databases 7

Table S4: Summary of Vocabulary Tasks and Categorisation for Data Synthesis 12

Table S5: Characteristics and Results of "Near Miss' Studies 14

Table S6: Authors Contacted for Additional Information 16

Table S7: Risk of Bias Details for Included Studies 17

Figure S1: Forest Plot for Receptive Vocabulary Tasks 19

Table S8: Results of Meta-Regression for Receptive Vocabulary Tasks 20

Figure S2: Forest Plot for Expressive Vocabulary Tasks 21

Figure S3: Funnel Plots from Meta-Analyses 22

Table S9: Results of Meta-Regression for Concurrent Array Tasks 23

References 24

Table S1

PRISMA Checklist

**Section & Topic**	**#**	**Checklist Item**	**Page #**
**TITLE**			
Title	1	Identify the report as a systematic review.	1
**ABSTRACT**			
Abstract	2	N/A	2
**INTRODUCTION**			
Rationale	3	Describe the rationale for the review in the context of existing knowledge.	3-9
Objectives	4	Provide an explicit statement of the objective(s) or question(s) the review addresses.	9-10
**METHODS**			
Eligibility criteria	5	Specify the inclusion and exclusion criteria for the review and how studies were grouped for the syntheses.	10-16
Information sources	6	Specify all databases, registers, websites, organisations, reference lists and other sources searched or consulted to identify studies. Specify the date when each source was last searched or consulted.	10
Search strategy	7	Present the full search strategies for all databases, registers and websites, including any filters and limits used.	11<br>Table S3
Selection process	8	Specify the methods used to decide whether a study met the inclusion criteria of the review, including how many reviewers screened each record and each report retrieved, whether they worked independently, and if applicable, details of automation tools used in the process.	11-13
Data collection process	9	Specify the methods used to collect data from reports, including how many reviewers collected data from each report, whether they worked independently, any processes for obtaining or confirming data from study investigators, and if applicable, details of automation tools used in the process.	12-13
Data Items	10a	List and define all outcomes for which data were sought. Specify whether all results that were compatible with each outcome domain in each study were sought (e.g., for all measures, time points, analyses), and if not, the methods used to decide which results to collect.	14-16
	10b	List and define all other variables for which data were sought (e.g., participant and intervention characteristics, funding sources). Describe any assumptions made about any missing or unclear information.	14-16
Study risk of bias assessment	11	Specify the methods used to assess risk of bias in the included studies, including details of the tool(s) used, how many reviewers assessed each study and whether they worked independently, and if applicable, details of automation tools used in the process.	13-14
Effect measures	12	Specify for each outcome the effect measure(s) (e.g., risk ratio, mean difference) used in the synthesis or presentation of results.	15
Synthesis methods	13a	Describe the processes used to decide which studies were eligible for each synthesis.	14-15
	13b	Describe any methods required to prepare the data for presentation or synthesis, such as handling of missing summary statistics, or data conversions.	16

	13c	Describe any methods used to tabulate or visually display results of individual studies and syntheses.	16-17
	13d	Describe any methods used to synthesize results and provide a rationale for the choice(s). If meta-analysis was performed, describe the model(s), method(s) to identify the presence and extent of statistical heterogeneity, and software package(s) used.	16-17
	13e	Describe any methods used to explore possible causes of heterogeneity among study results.	16-17
	13f	Describe any sensitivity analyses conducted to assess robustness of the synthesized results.	N/A
Reporting bias assessment	14	Describe any methods used to assess risk of bias due to missing results in a synthesis (arising from reporting biases).	17
Certainty assessment	15	Describe any methods used to assess certainty (or confidence) in the body of evidence for an outcome.	N/A
**RESULTS**			
Study selection	16a	Describe the results of the search and selection process, from the number of records identified in the search to the number of studies included in the review, ideally using a flow diagram.	17-18 Figure 1
	16b	Cite studies that met many but not all inclusion criteria ('near-misses') and explain why they were excluded.	18 Table S5
Study characteristics	17	Cite each included study and present its characteristics.	20-22 Tables 3 & 4
Risk of bias in studies	18	Present assessments of risk of bias for each included study.	19-20 Tables 2 & S7
Results of individual studies	19	For all outcomes, present, for each study: (a) summary statistics for each group (where appropriate) and (b) an effect estimate and its precision (e.g. confidence/credible interval), ideally using structured tables or plots.	Tables 3, 4, & S5 Figures 3-6
Results of syntheses	20a	For each synthesis, briefly summarise the characteristics and risk of bias among contributing studies.	22-29
	20b	Present results of all statistical syntheses conducted. If meta-analysis was done, present for each the summary estimate and its precision (e.g. confidence/credible interval) and measures of statistical heterogeneity. If comparing groups, describe the direction of the effect.	22-29
	20c	Present results of all investigations of possible causes of heterogeneity among study results.	22-29
	20d	Present results of all sensitivity analyses conducted to assess the robustness of the synthesized results.	25-27
Reporting biases	21	Present assessments of risk of bias due to missing results (arising from reporting biases) for each synthesis assessed.	22-29 Figure S3
Certainty of evidence	22	Present assessments of certainty (or confidence) in the body of evidence for each outcome assessed.	N/A
**DISCUSSION**			
Discussion	23a	Provide a general interpretation of the results in the context of other evidence.	29-34
	23b	Discuss any limitations of the evidence included in the review.	29-37
	23c	Discuss any limitations of the review processes used.	34-37

	23d	Discuss implications of the results for practice, policy, and future research.	37-38
**OTHER**			
Registration and protocol	24a	Provide registration information for the review, including register name and registration number, or state that the review was not registered.	10
	24b	Indicate where the review protocol can be accessed, or state that a protocol was not prepared.	10
	24c	Describe and explain any amendments to information provided at registration or in the protocol.	Table S2
Support	25	Describe sources of financial or non-financial support for the review, and the role of the funders or sponsors in the review.	1
Competing interests	26	Declare any competing interests of review authors.	1
Availability of data, code, and other materials	27	Report which of the following are publicly available and where they can be found: template data collection forms; data extracted from included studies; data used for all analyses; analytic code; any other materials used in the review.	1

Note. Page numbers referred to the original, unformatted version.

Table S2

Summary of Amendments to PROSPERO Protocol (CRD42019125132; Pickering et al., 2019)

No.	Date Published; Stage	Description
1	26 June 2019; Screening	Updated progress/status. No other changes were made.
2	08 October 2019; Screening	Updated progress/status. No other changes were made.
3	22 October 2020; Data Extraction	1. Addition of a new author (who will also serve as a co-reviewer) - the initial main co-reviewer is no longer able to complete some parts of the review, and thus a new co-reviewer was needed.<br>2. Revision of funding sources<br>3. Slight revision to exclusion criteria - the first exclusion criteria under question 19 has been revised to exclude children with medical conditions (as well as neurodevelopmental conditions). This was added as research on these children was appearing within the systematic search, however, as the aim of this review is to understand visual memory and vocabulary relations within typical development, it was decided to exclude these children as well, as the impact of their medical conditions (e.g., tumours, cancer) on either visual memory or vocabulary is unclear.
4	12 March 2021; Data Analysis	1. Revision to author list; addition of new author (primary co-reviewer, JP), and removal of two previous authors who were unable to continue working on the project.

		2. Updated strategy for data synthesis to include new grouping method (memory tasks, rather than vocabulary tasks), and details of planned meta-analyses.<br>3. Updated type and method of review to include meta-analysis.<br>4. Updated progress/status.
5	12 March 2022; Submission	1. Revised expected completion date.<br>2. Updated progress/status.

Table S3

Full Search Strategy for all Databases

**Database/s (*n*)**	**Line No.**	**Terms**
Cochrane Library Database (*n* = 728)	1	visu*
	2	spati*
	3	visu*spati
	4	spati*visu*
	5	“visu* spati*”
	6	#1 OR #2 OR #3 OR #4 OR #5
	7	MeSH descriptor: [Memory] explode all trees
	8	MeSH descriptor: [Memory, Short-Term] explode all trees
	9	MeSH descriptor: [Memory, Short-Term] explode all trees
	10	MeSH descriptor: [Memory, Long-Term] explode all trees
	11	memory
	12	"short term memory"
	13	“working memory”
	14	“complex memory”
	15	“declarative memory”
	16	“long term memory”
	17	STM
	18	LTM
	19	WM
	20	#7 OR #8 OR #9 OR #10 OR #11 OR #12 OR #13 OR #14 OR #15 OR #16 OR #17 OR #18 OR #19
	21	#6 AND #20
	22	MeSH descriptor: [Vocabulary] explode all trees
	23	MeSH descriptor: [Comprehension] explode all trees
	24	vocabulary
	25	“mental lexicon”
	26	lexicon
	27	“word knowledge”
	28	comprehension
	29	words
	30	#22 OR #23 OR #24 OR #25 OR #26 OR #27 OR #28 OR #29
	31	child*
	32	MeSH descriptor: [Child] explode all trees
	33	MeSH descriptor: [Child, Preschool] explode all tree
	34	MeSH descriptor: [Child Language] explode all trees
	35	#31 OR #32 OR #33 OR #34
	36	#21 AND #30
	37	#36 AND #35

ERIC (ProQuest)	S1	noft(visu*)
(*n* = 703)	S2	noft(spati*)
	S3	noft(visu* spati*)
	S4	noft(spati* visu*)
	S5	S1 or S2 OR S3 OR S4
	S6	MAINSUBJECT.EXACT.EXPLODE("Long Term Memory") OR MAINSUBJECT.EXACT.EXPLODE("Short Term Memory") OR MAINSUBJECT.EXACT.EXPLODE("Memory")
	S7	noft("memory")
	S8	noft("short term memory")
	S9	noft("working memory")
	S10	noft("complex memory")
	S11	noft("declarative memory")
	S12	noft("long term memory")
	S13	noft("STM")
	S14	noft("WM")
	S15	noft("LTM")
	S16	S6 OR S7 OR S8 OR S9 OR S10 OR S11 OR S12 OR S13 OR S14 OR S15
	S17	S5 AND S16
	S18	(MAINSUBJECT.EXACT.EXPLODE("Vocabulary Development") OR MAINSUBJECT.EXACT.EXPLODE("Vocabulary Skills") OR MAINSUBJECT.EXACT.EXPLODE("Vocabulary")) OR MAINSUBJECT.EXACT.EXPLODE("Comprehension")
	S19	noft("vocabulary")
	S20	noft("mental lexicon")
	S21	noft("lexicon")
	S22	noft("word knowledge")
	S23	noft("comprehension")
	S24	noft("words")
	S25	S18 OR S19 OR S20 OR S21 OR S22 OR S23 OR S24
	S26	noft(child*)
	S27	MAINSUBJECT.EXACT.EXPLODE("Children") OR MAINSUBJECT.EXACT.EXPLODE("Child Language")
	S28	S26 OR S27
	S29	S17 AND S25
	S30	S28 AND S29
Ovid Databases:	1	visu*.mp
	2	spati*.mp
EMBASE	3	visu* spati*.mp
(1974-present)	4	spati* visu*.mp
(*n* = 1,209)	5	1 or 2 or 3 or 4

	6	memory.mp
Medline	7	"short term memory".mp
(1946-present)	8	"working memory".mp
(*n* = 942)	9	"complex memory".mp
	10	"declarative memory".mp
PsychINFO	11	"long term memory".mp
(1806-present)	12	"STM".mp
(*n* = 1,615)	13	"WM".mp
	14	"LTM".mp
Note. mp=title, abstract, heading word, table of contents, key concepts, original title, tests & measures	15	exp LONG TERM MEMORY/ or exp VISUOSPATIAL MEMORY/ or exp SHORT TERM MEMORY/ or exp VISUAL MEMORY/ or exp MEMORY/ or exp SPATIAL MEMORY/
	16	6 or 7 or 8 or 9 or 10 or 11 or 12 or 13 or 14 or 15
	17	5 and 16
	18	vocabulary.mp
	19	"mental lexicon".mp
	20	lexicon.mp
	21	"word knowledge".mp
	22	"comprehension".mp
	23	words.mp
	24	exp Mental Lexicon/
	25	exp VOCABULARY/
	26	exp "WORDS (PHONETIC UNITS)"/ or exp Vocabulary/
	27	18 or 19 or 20 or 21 or 22 or 23 or 24 or 25 or 26
	28	child*.mp
	29	17 and 27
	30	28 and 29
PudMed	1	visu*
(*n* = 1,192)	2	spati*
	3	"visu* spati*"
	4	"spati* visu*"
	5	(#1 OR #2 OR #3 OR #4)
	6	memory
	7	"short term memory"
	8	"working memory"
	9	"complex memory"
	10	"declarative memory"
	11	"long term memory"
	12	"STM"
	13	“LMT”
	14	“WM”
	15	(#6 OR #7 OR #8 OR #9 OR #10 OR #11 OR #12 OR #13 OR #14)

	16	(#5 and #15)
	17	"vocabulary"
	18	"mental lexicon"
	19	"lexicon"
	20	"word knowledge"
	21	"comprehension"
	22	"words"
	23	(#17 or #18 or #19 OR #20 OR #21 OR #22)
	24	child*
	25	childh*
	26	childr*
	27	(#24 OR #25 OR #26)
	28	(#16 AND #23)
	29	(#27 AND #28)
Scopus (Elsevier) (*n* = 1,680)	N/A	(TITLE-ABS-KEY (child*)) AND ((((TITLE-ABS-KEY (memory)) OR (TITLE-ABS-KEY ("short term memory")) OR (TITLE-ABS-KEY ("working memory")) OR (TITLE-ABS-KEY ("complex memory")) OR (TITLE-ABS-KEY ("declarative memory")) OR (TITLE-ABS-KEY ("long term memory")) OR (TITLE-ABS-KEY ("STM")) OR (TITLE-ABS-KEY ("LTM")) OR (TITLE-ABS-KEY ("WM")))) AND ((TITLE-ABS-KEY (visu*)) OR (TITLE-ABS-KEY (spati*)) OR (TITLE-ABS-KEY ("visu* spati*")) OR (TITLE-ABS-KEY ("spati* visu*"))))) AND ((TITLE-ABS-KEY ("vocabulary")) OR (TITLE-ABS-KEY ("mental lexicon")) OR (TITLE-ABS-KEY ("lexicon")) OR (TITLE-ABS-KEY ("word knowledge")) OR (TITLE-ABS-KEY ("comprehension")) OR (TITLE-ABS-KEY ("words")))))
Web of Science (ISI) (*n* = 2,082)	1	**TOPIC:** (visu*)
	2	**TOPIC:** (spati*)
	3	**TOPIC:** ("visu* spati*")
	4	**TOPIC:** ("spati* visu*")
	5	#4 OR #3 OR #2 OR #1
	6	**TOPIC:** (memory)
	7	**TOPIC:** ("short term memory")
	8	**TOPIC:** ("working memory")
	9	**TOPIC:** ("complex memory")
	10	**TOPIC:** ("declarative memory")
	11	**TOPIC:** ("long term memory")

12	**TOPIC:** ("LTM")
13	**TOPIC:** ("STM")
14	**TOPIC:** ("WM")
15	#14 OR #13 OR #12 OR #11 OR #10 OR #9 OR #8 OR #7 OR #6
16	#15 AND #5
17	**TOPIC:** ("vocabulary")
18	**TOPIC:** ("mental lexicon")
19	**TOPIC:** ("lexicon")
20	**TOPIC:** ("word knowledge")
21	**TOPIC:** ("comprehension")
22	**TOPIC:** ("words")
23	**TOPIC:** ("child*")
24	#22 OR #21 OR #20 OR #19 OR #18 OR #17
25	#24 AND #16
26	#25 AND #23

Table S4

Summary of Vocabulary Tasks and Categorisation for Data Synthesis

Task Name; Citation	Task Description	Modality	Domain	Citation/s in Review
Arabic Comprehension Test of Abstract Words (ACTAW); Batnini and Uno (2015)	Participants much choose which of 6 presented pictures matches a spoken abstract word.	Receptive	Visual	Batnini and Uno (2015)
British Picture Vocabulary Scale (BPVS); Dunn et al. (1997)	Participants must choose which of 4 presented pictures matches a spoken word.	Receptive	Visual	Adams et al. (1999); Critten et al. (2018); Laws (2002); Michas and Henry (1994)
Expressive One-Word Picture Vocabulary Test (EOWPVT); Brownell (2000a)	Participants must provide a one-word definition of a presented picture.	Expressive	Visual	Stokes et al. (2017)
Expressive Vocabulary; Cornu et al. (2018)	Participants are presented with 18 pictures of objects and asked to name each object.	Expressive	Visual	Cornu et al. (2018)
Expressive Vocabulary subtest, CELF-4; Semel et al. (2006)	Participants are presented with pictures of actions (e.g., colouring) or objects (e.g., saxophone) and must provide the verbal label.	Expressive	Visual	Malone et al. (2020)
Peabody Picture Vocabulary Test (PPVT); Dunn and Dunn (2007)	Participants must choose which of 4 presented pictures matches a spoken word.	Receptive	Visual	Barbosa et al. (2017); Evans et al. (2008); Meneghetti et al. (2020); Montoya et al. (2019); Obeid and Brooks (2018); Séguin et al. (2009); Williams et al. (1977)
Picture Naming (WPPSI); Wechsler (2016)	Participants are presented with 18 pictures and are asked to identify each image.	Expressive	Visual	Veraksa et al. (2018)
Picture Vocabulary (WJ-III); Woodcock et al. (2007)	Participants are presented with pictures of objects ranging from common to specialised	Expressive	Visual	Vukovic and Lesaux (2013)

	(e.g., star, gavel), and asked to name the object.			
Receptive & Expressive Vocabulary Test (REVT); Kim et al. (2009)	REVT-R (Receptive): Participants select a picture that matches a spoken target word.	Receptive	Visual	Yoo and Yim (2018)
	REVT-E (Expressive): Participants must name a presented picture.	Expressive	Visual	
Receptive One-Word Picture Vocabulary Test (ROWPVT); Brownell (2000b)	Participants must choose which of 4 presented pictures matches a spoken word.	Receptive	Visual	Stokes et al. (2017)
Synonym Subtest (California Reading Test); Claes et al. (1967)	Participants read a target word and select which of 5 written words has "the same or almost the same" meaning.	Receptive	Verbal	Seigneuric et al. (2000)
Toets Tweetaligheid (TT) [Test for Bilingualism]; Verhoeven et al. (1995) & Taaltoets Alle Kinderen (TAK) [Language Test for All Children]; Verhoeven and Vermeer (2002)[a]	Participants must choose which of 4 line drawings matches a spoken word	Receptive	Visual	Blom et al. (2014)
Verbal Meaning Test; Resing et al. (2012)	Participants must choose which of four figures match a spoken word.	Receptive	Visual	van der Graaf et al. (2016)
Vocabulary (WASI; WISC-IV); Wechsler (2011a, 2011b)	Participants are presented with a spoken word and asked to provide a definition.	Expressive	Verbal	Palombo and Cuadro (2020); Senese et al. (2020); Wilson et al. (2018)
Vocabulary Definitions; Bock et al. (2015)	Participants must provide oral definitions for 10 words (tractor, bread, chain, harp, bonnet, spinach, dentist, panda, cream, president).	Expressive	Verbal	Bock et al. (2015)
Wortschataztest Synonym Test (Culture Fair Intelligence Test); Weiss (1991)	Participants are presented with a target word and select which of 5 written words has the same meaning (or closest too).	Receptive	Verbal	Studer-Luethi et al. (2016)

Note. WPPSI = Wechsler Preschool and Primary Scale of Intelligence; WJ-III = Woodcock-Johnson-III; WASI = Wechsler Abbreviated Scale of Intelligence; WISC = Wechsler Intelligence Scale for Children. [a] Tests combined for one measure.

Table S5

Characteristics and Results of "Near Miss' Studies

Study Details				Participants		Measures		Results		
**#**	**Citation**	**Language**	**Aim Relevant?**	**N**	**Age M (SD); Range**	**Memory Task/s**	**Vocabulary Task/s**	**Comparison**	**Statistic, *p***	**Fishers' *z* [91% CI]**
1	Alloway and Elsworth (2012)	English	Y	1. High IQ (n = 44)<br>2. Av IQ (n = 38)	1. 10.4 (1.5)<br>2. 9.8 (1.0)	AWMA Spatial Recall	WASI Vocabulary	Pearson correlations, standardised (age-adjusted) scores	1. r = .22, *ns*<br>2. r = .24, *ns*	1. 0.22 [-0.08, 0.52]<br>2. 0.25 [-0.09, 0.57]
2	Alloway and Passolunghi (2011)	Italian	N	206	7.96 (0.3)	1. AWMA STM: Dot Matrix, Mazes Memory, Block Recall<br>2. AWMA WM: Odd-One-Out, Mr X., Spatial Recall	PMA Vocabulary Subtest	Pearson correlation b/w AWMA STM and WM composites with raw PMA scores	1. r = .53, <.005<br>2. r = .50, <.005	1. 0.59 [0.45, 0.72]<br>2. 0.55 [0.42, 0.68]
3	DeNigris and Brooks (2018)	English	N	62	8.17 (1.25); 6.0-10.67	One-Shape Array Memory Task	PPVT-IV	Partial correlation (controlling for age)	r = .28, < .05	0.29 [0.03, 0.54]
4	Henry and Maclean (2003)	English	Y	1. 45<br>2. 41	1. 12.08 (0.38); 11.33-12.92<br>2. 7.92 (0.45); 7.0-8.92	Spatial Span & Pattern Span	BAS Word Definition	Pearson correlation between BAS "ability" scores and a composite of	1. r = -.04, *ns*<br>2. r = .43, <.01	1. -0.04 [-0.34, 0.26]<br>2. 0.46 [0.14, 0.78]

							the memory tasks		
5	Hooper et al. (2011)	English	N	104[a]	6.0-7.33	1. WRAML Picture Memory (Immediate)<br>2. WRMAL Picture Memory (Recognition)<br>3. WISC-IV-I Spatial Span (Forward)<br>4. WISC-IV-I Spatial Span (Backward)	PPVT-IV	Pearson correlations using *z*-scores	1. *r* = .362<br>2. *r* = .016<br>3. *r* = .163<br>4. *r* = .358	1. 0.38 [0.18, 0.57]<br>2. 0.02 [-0.18, 0.21]<br>3. 0.165 [-0.03, 0.36]<br>4. 0.37 [0.18, 0.57]
6	Joseph et al. (2005)	English	Y	31	8.25 (2.08); 5.08-11.67	1. Block Span (Forward)<br>2. Block Span (Backward)	PPVT-III & EVT	Partial correlation (controlling for non-verbal intelligence) between combined vocabulary score and memory scores	1. *r* = .02, *ns*<br>2. *r* = .06, *ns*	1. 0.02 [-0.35, 0.39]<br>2. 0.06 [-0.31, 0.43]
7	Lum et al. (2012)	English	Y	51	9.85 (0.7); 8.5-11.41	1. STM: WMTB-C Mazes Memory, Block Recall<br>2. DM: CMS Dot Locations, Faces	EOWPVT & ROWPVT	Pearson correlation with composite memory measures (STM, DM) and composite	1. *r* = -.029, *ns*<br>2. *r* = .251, *ns*	1. -0.03 [-0.32, 0.25]<br>2. 0.26 [-0.03, 0.54]

								vocabulary measure		
8	Metcalfe and Stratford (1986)	English	N	162	5.66 (1.42); 3.5-8.2	Custom Task: recall for a sequence of 3 different-sized red squares	PPVT	Pearson correlation with PPVT expressed as mental age	*r* = .40, <.001	0.42 [0.26, 0.58
9	Rasmussen et al. (2009)	English	N	28	6.32 (0.87); 4.17-8.75	WMTB-C Block Span	PPVT-III	Partial correlation (controlling for age)	*r* = -.08, *ns*	-0.08 [-0.47, 0.31]
10	Vukovic et al. (2014)	English	N	163[b]	6.5 (0.3)	WMTB-C Mazes Memory & Block Recall	WASI Vocabulary	Pearson correlation with composite of memory tasks	*r* = .32, <.001	0.33 [0.18, 0.49]

Note. AWMA = Automated Working Memory Assessment; WASI = Weschler Abbreviated Scale of Intelligence; STM = Short-Term Memory; WM = Working Memory; PMA = Primary Mental Ability (*Batteria Primaria di Abilità*); PPVT = Peabody Picture Vocabulary Test; BAS = British Ability Scale; WRAML = Wide Range Assessment of Memory and Learning – 2; WISC-IV-I = Weschler Intelligence Scale for Children – Fourth Edition – Integrated); EVT = Expressive Vocabulary Test; WMTB-C = Working Memory Test Battery for Children; DM = Declarative Memory; CMS = Children's Memory Scale; EOWPVT = Expressive One-Word Picture Vocabulary Test; ROWPVT = Receptive One-Word Picture Vocabulary Test.

[a] Used data from Cohort 1, Grade 1. [b] Used first grade results

Table S6

Correspondence for Additional Study Information

**1. Evans et al. (2008) - Preschoolers' Attention to Print During Shared Book Reading**	
Question	Please indicate the mean (and SD) age of participants (at the first point of testing) for the whole sample (n = 76).
Response	The average age of all the children was 4.44, SD 9.56.
**2. Montoya et al. (2019) - Executive function in Chilean preschool children: Do short-term memory, working memory, and response inhibition contribute differentially to early academic skills?**	
Question	Can you please clarify if the M and SD provided for participant ages (section 2.1, page 190) is represented in years, or months?
Response	The participant ages and their descriptive are represented in years.
**3. Palombo and Cuadro (2020) - The incidence of visual-motor processes in the acquisition of orthographic representations in Spanish-speaking schoolchildren (La incidencia de los procesos perceptivo-motrices en la adquisición de las representaciones ortográficas en escolares hispanoparlante)**	
Questions	1. Can you please confirm the total n included in the study? The text (*Participants*, page 492) states 96, but when I add up the numbers provided in Table 1, this comes to 97.<br>2. Can you please confirm if the correlations provided in Table 3 are for the whole sample (i.e., n = 96/97).
Responses	1. The number of participants is 97.<br>2. Yes, the correlation was performed for the total sample.
**4. Wilson et al. (2018) - Executive function in middle childhood and the relationship with theory of mind**	
Question	Please indicate the mean (and SD) age of participants for the whole sample (n = 126).
Response	No response received.
**5. Yoo and Yim (2018) - Relationship among Executive Functions, Vocabulary and Reading Skills in School-Aged Children with and without Poor Vocabulary**	
Question	Can you please provide a brief description of what participants were required to do for both the receptive and expressive subtests of the REVT? (i.e., select a picture that matches a spoken word, name presented pictures, provide definitions of common words, etc).
Response	No response received.

Table S7

Risk of Bias Details for Included Studies

Study Details		Intro	Method									Results						Discussion		Other		Total
#	Citation	1:Aims	2: Study Design	3: Sample Size Justification	4: Target Population	5: Sampling Frame	6: Sample Selection	7: Non-Responders	8: Appropriate Measurement	9: Reliable Measurement	10: Statistical Significance	11: Repeatability	12: Basic Data	13: Response Rate	14: Non-Responders	15: internal Consistency	16: All Results Presented	17: Conclusions Justified	18: Limitations Discussed	19: Funding a& COI Declared	20: Ethics & Consent Obtained	%
1	Adams et al. (1999)	Green	Green	Red	Green	Green	Green	Yellow	Green	Yellow	Yellow	Green	Green	Black	Black	Green	Green	Green	Green	Red	Red	75%
2	Barbosa et al. (2017)	Green	Green	Red	Green	Green	Yellow	Yellow	Yellow	Yellow	Red	Green	Green	Yellow	Black	Green	Green	Green	Green	Green	Green	76%
3	Batnini and Uno (2015)	Green	Green	Red	Green	Green	Yellow	Yellow	Green	Yellow	Green	Green	Yellow	Yellow	Black	Green	Green	Green	Green	Red	Green	76%
4	Blom et al. (2014)	Green	Green	Red	Green	Green	Red	Green	Green	Yellow	Yellow	Green	Green	Green	Green	Green	Green	Green	Green	Yellow	Red	78%
5	Bock et al. (2015)	Green	Green	Red	Yellow	Yellow	Yellow	Yellow	Green	Red	Yellow	Green	Yellow	Yellow	Yellow	Yellow	Green	Green	Green	Yellow	Red	60%
6	Cornu et al. (2018)	Green	Green	Red	Green	Green	Red	Yellow	Green	Red	Green	Green	Green	Green	Red	Green	Green	Green	Green	Yellow	Green	75%
7	Critten et al. (2018)	Green	Green	Red	Green	Green	Red	Yellow	Green	Green	Yellow	Green	Green	Yellow	Black	Green	Green	Green	Red	Yellow	Green	74%
8	Evans et al. (2008)	Green	Green	Red	Green	Green	Green	Green	Green	Green	Yellow	Green	Green	Red	Red	Red	Green	Green	Green	Yellow	Yellow	76%
9	Laws (2002)	Green	Green	Red	Green	Yellow	Red	Yellow	Green	Yellow	Yellow	Green	Green	Yellow	Black	Green	Green	Green	Green	Red	Red	66%
10	Malone et al. (2020)	Green	Green	Red	Red	Yellow	Yellow	Yellow	Green	Green	Yellow	Yellow	Yellow	Green	Red	Green	Green	Green	Green	Yellow	Green	68%
11	Meneghetti et al. (2020)	Green	Green	Red	Green	Green	Yellow	Yellow	Green	Yellow	Green	Green	Green	Yellow	Green	Green	Green	Green	Green	Green	Green	84%
12	Michas and Henry (1994)	Green	Green	Red	Green	Yellow	Red	Green	Green	Yellow	Yellow	Green	Yellow	Red	Red	Green	Green	Green	Green	Red	Red	60%

13	Montoya et al. (2019)	Green	Green	Red	Green	Green	Green	Orange	Green	Green	Green	Green	Green	Orange	Black	Green	Green	Green	Green	Orange	Green	87%
14	Obeid and Brooks (2018)	Green	Green	Red	Green	Green	Green	Orange	Green	Green	Orange	Green	Green	Orange	Black	Green	Green	Green	Orange	Green	Green	84%
15	Palombo and Cuadro (2020)	Green	Green	Red	Orange	Orange	Orange	Orange	Green	Orange	Orange	Green	Green	Orange	Black	Green	Green	Green	Red	Orange	Orange	66%
16	Séguin et al. (2009)	Green	Green	Red	Green	Green	Green	Green	Green	Orange	Orange	Green	Orange	Green	Green	Green	Green	Green	Green	Green	Green	88%
17	Seigneuric et al. (2000)	Green	Green	Red	Orange	Orange	Orange	Orange	Green	Orange	Orange	Green	Orange	Orange	Black	Green	Green	Green	Green	Red	Red	63%
18	Senese et al. (2020)	Green	Green	Red	Green	Green	Red	Orange	Green	Green	Green	Orange	Orange	Orange	Black	Green	Green	Green	Green	Orange	Green	76%
19	Stokes et al. (2017)	Green	Green	Red	Green	Green	Green	Orange	Green	Orange	Orange	Green	Green	Orange	Black	Green	Green	Green	Green	Orange	Green	82%
20	Studer-Luethi et al. (2016)	Green	Green	Red	Green	Green	Orange	Orange	Green	Orange	Orange	Green	Orange	Orange	Black	Green	Green	Green	Green	Red	Orange	71%
21	van der Graaf et al. (2016)	Green	Green	Red	Green	Orange	Orange	Orange	Green	Green	Green	Green	Orange	Orange	Black	Green	Green	Green	Green	Orange	Orange	76%
22	Veraksa et al. (2018)	Green	Green	Red	Green	Green	Orange	Orange	Green	Green	Orange	Orange	Orange	Orange	Black	Green	Green	Green	Green	Orange	Green	76%
23	Vukovic and Lesaux (2013)	Green	Green	Red	Green	Green	Orange	Green	Green	Green	Orange	Green	Green	Green	Red	Green	Green	Green	Green	Orange	Red	78%
24	Williams et al. (1977)	Green	Green	Red	Green	Green	Green	Orange	Green	Green	Orange	Orange	Orange	Orange	Black	Green	Red	Green	Red	Red	Red	61%
25	Wilson et al. (2018)	Green	Green	Red	Green	Green	Red	Orange	Green	Green	Orange	Green	Orange	Orange	Black	Green	Green	Green	Green	Orange	Green	76%
26	Yoo and Yim (2018)	Green	Green	Red	Green	Green	Orange	Orange	Green	Orange	Orange	Green	Green	Orange	Black	Green	Green	Green	Green	Orange	Red	74%

Note. Green = yes; Red = no; Orange = unclear; Black = not applicable; Intro = Introduction; COI = Conflict of Interest. For details of each criteria, see supplemental documentation from Downes et al. (2016).

Figure S1

Forest Plot Showing Pooled Correlations (Fisher's Z) for Receptive Vocabulary Tasks

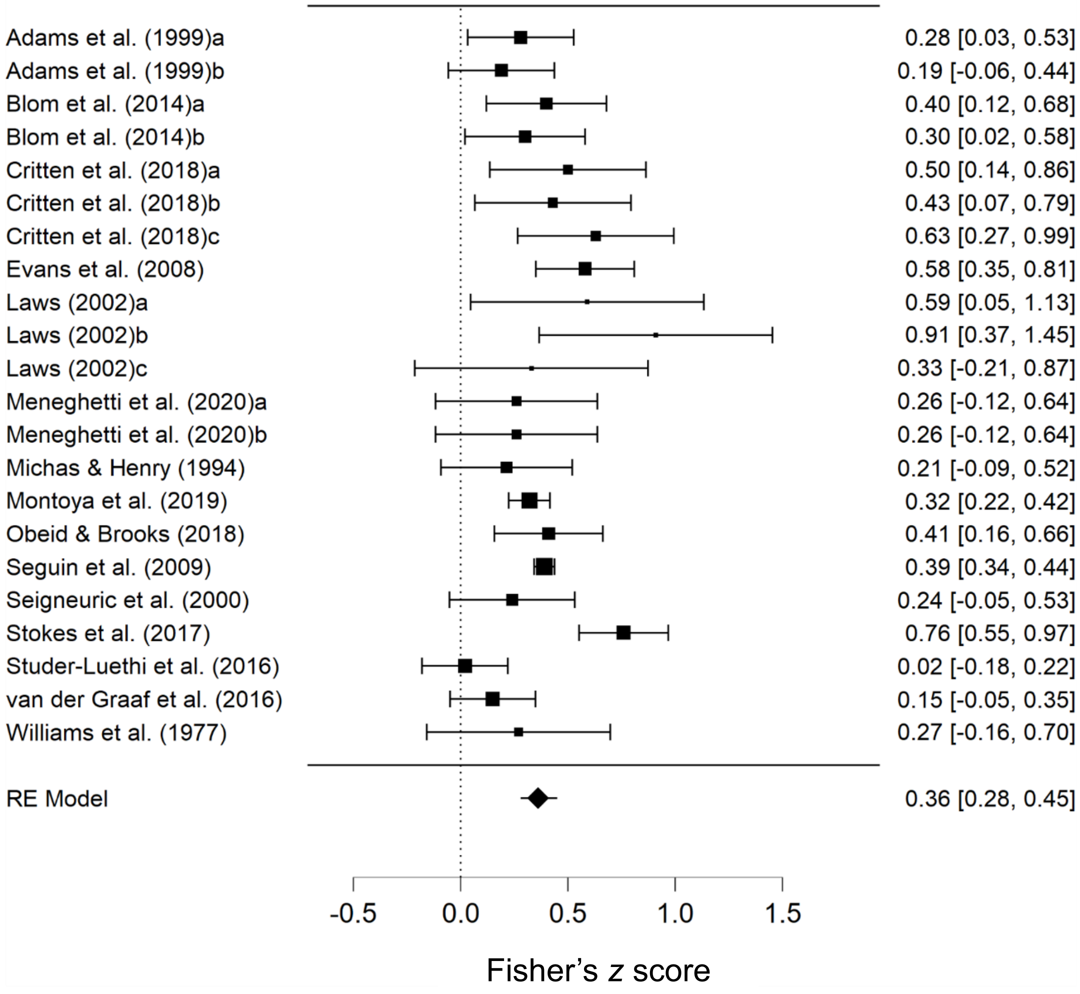

Note. RE = Random Effects.

Table S8

Results of Meta-Regression for Receptive Vocabulary Tasks

**Random Effects Model**				
	**Estimate**	**SE**	**Z**	***p***
Intercept	-1.190	1.512	-0.787	0.431
Age (Mean)	0.147	0.170	0.863	0.388
Vocabulary Domain	1.570	1.519	1.033	0.301
Age * Vocabulary Domain	-0.145	0.172	-0.842	0.400
**Heterogeneity**				
**Q**	**Df**	***p***	**τ^2**	**I^2 (%)**
34.496	18	0.011	0.016	56.46

Note. SE = Standard Error; df = degrees of freedom.

Figure S2

Forest Plot Showing Pooled Correlations (Fisher's Z) for Expressive Vocabulary Tasks

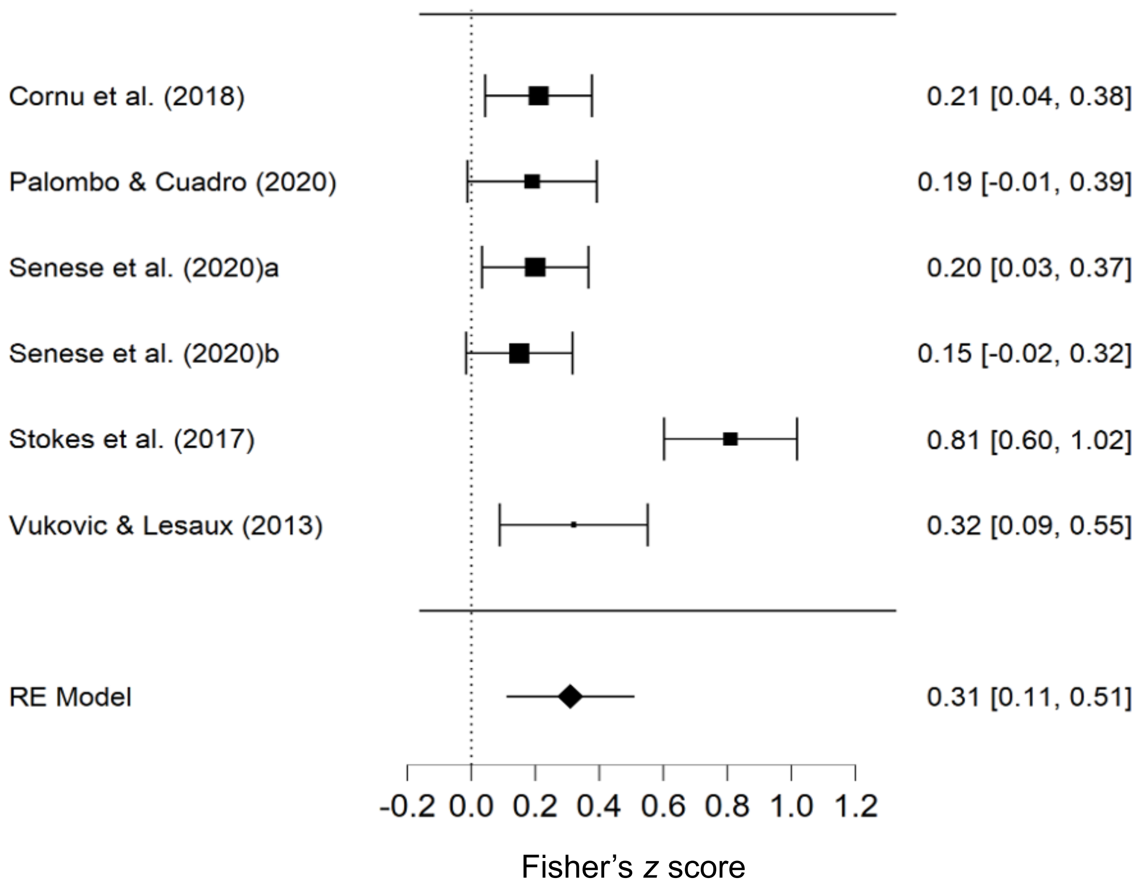

Note. RE = Random Effects.

Figure S3

Funnel Plots from Temporal-Span, Concurrent Array, and Executive Judgement Meta-Analyses

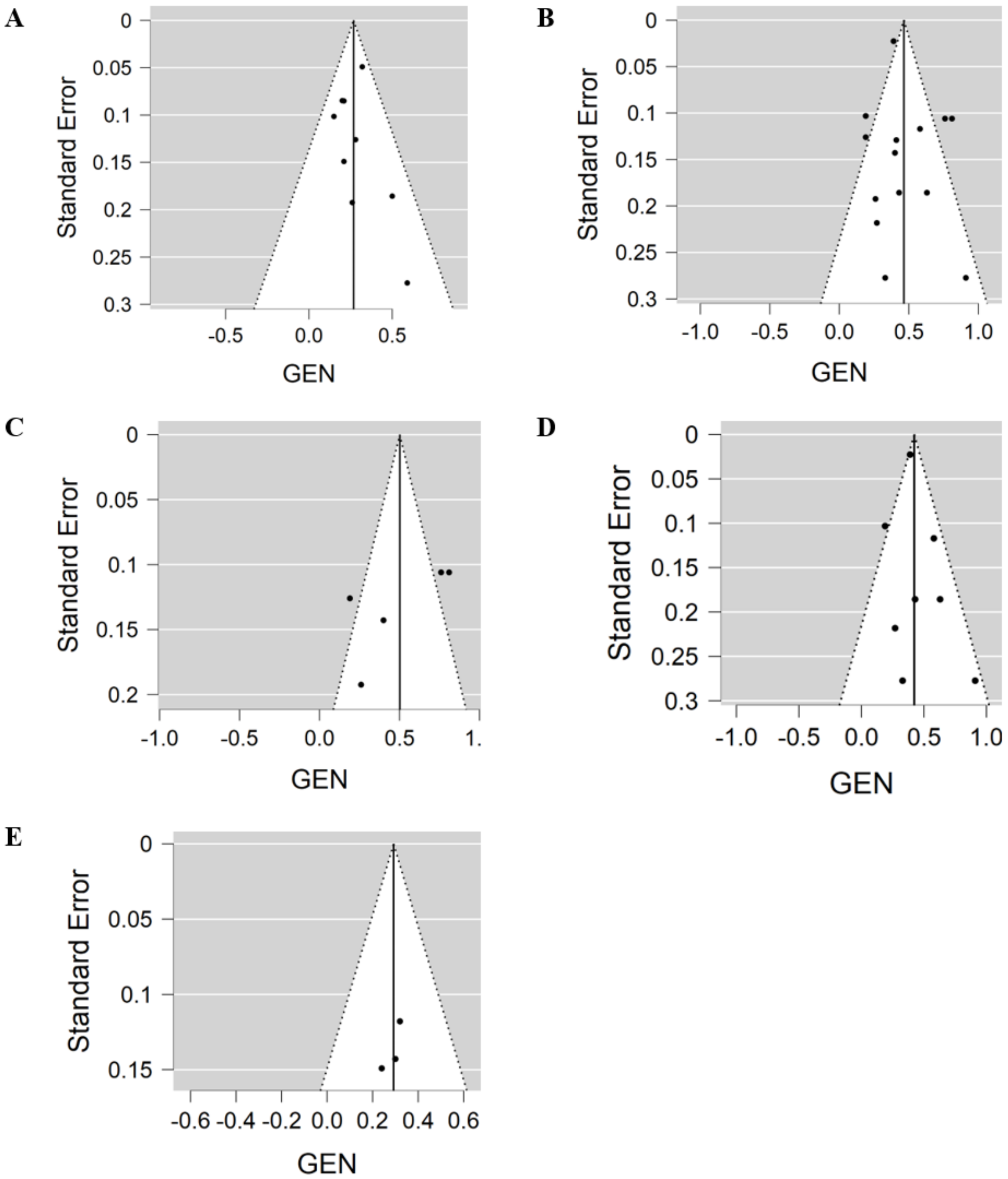

Note. Panel A: Funnel plot for temporal-span tasks. Panel B: Funnel plot for concurrent array tasks (all). Panel C: Funnel plot for spatial concurrent array tasks. Panel D: Funnel plot for visuo-perceptual concurrent array tasks. Panel E: Funnel plot for executive judgement tasks.

Table S9

Results of Meta-Regression for Concurrent Array Tasks

**Random Effects Model**				
	**Estimate**	**SE**	**Z**	***p***
Intercept	1.184	0.305	3.878	< .001
Age (Mean)	-0.102	0.041	-2.468	0.014
Vocabulary Modality	-0.650	0.358	-1.813	0.070
Age * Vocabulary Modality	0.087	0.053	1.657	0.097
**Heterogeneity**				
**Q**	**df**	***p***	**τ^2**	**I^2 (%)**
22.370	10	.013	.021	54.22

Note. SE = Standard Error; df = degrees of freedom.

References

Adams, A. M., Bourke, L., & Willis, C. (1999). Working memory and spoken language comprehension in young children. *International Journal of Psychology, 34*(5-6), 364-373. https://doi.org/10.1080/002075999399701

Alloway, T. P., & Elsworth, M. (2012). An investigation of cognitive skills and behavior in high ability students. *Learning and Individual Differences*, *22*(6), 891-895. https://doi.org/10.1016/j.lindif.2012.02.001

Alloway, T. P., & Passolunghi, M. C. (2011). The relationship between working memory, IQ, and mathematical skills in children. *Learning and Individual Differences*, *21*(1), 133-137. https://doi.org/10.1016/j.lindif.2010.09.013

Barbosa, P. G., Jiang, Z., & Nicoladis, E. (2017). The role of working and short-term memory in predicting receptive vocabulary in monolingual and sequential bilingual children. *International Journal of Bilingual Education and Bilingualism*, 1-17. https://doi.org/10.1080/13670050.2017.1314445

Batnini, S., & Uno, A. (2015). Investigation of basic cognitive predictors of reading and spelling abilities in Tunisian third-grade primary school children. *Brain and Development, 37*(6), 579-591. https://doi.org/10.1016/j.braindev.2014.09.010

Blom, E., Küntay, A. C., Messer, M., Verhagen, J., & Leseman, P. (2014). The benefits of being bilingual: Working memory in bilingual Turkish–Dutch children. *Journal of Experimental Child Psychology, 128*, 105-119. https://doi.org/10.1016/j.jecp.2014.06.007

Bock, A. M., Gallaway, K. C., & Hund, A. M. (2015). Specifying links between executive functioning and theory of mind during middle childhood: Cognitive flexibility predicts social understanding. *Journal of Cognition and Development, 16*(3), 509-521. https://doi.org/10.1080/15248372.2014.888350

Brownell, R. (2000a). *Expressive One-Word Picture Vocabulary Test (2nd ed.)*. Academic Therapy Publications.

Brownell, R. (2000b). *Receptive One-Word Picture Vocabulary Test (2nd ed.)*. Academic Therapy Publications.

Claes, M., Dehant, A., Lamy, J., & Gille, A. (1967). *Test de lecture California [California reading test]*. Editest.

Cornu, V., Schiltz, C., Martin, R., & Hornung, C. (2018). Visuo-spatial abilities are key for young children's verbal number skills. *Journal of Experimental Child Psychology, 166*, 604-620. https://doi.org/10.1016/j.jecp.2017.09.006

Critten, V., Campbell, E., Farran, E., & Messer, D. (2018). Visual perception, visual-spatial cognition and mathematics: Associations and predictions in children with cerebral palsy. *Research in Developmental Disabilities, 80*, 180-191. https://doi.org/10.1016/j.ridd.2018.06.007

DeNigris, D., & Brooks, P. J. (2018). The role of language in temporal cognition in 6- to 10-year-old children. *Journal of Cognition and Development, 19*(4), 431-455. https://doi.org/10.1080/15248372.2018.1483372

Downes, M. J., Brennan, M. L., Williams, H. C., & Dean, R. S. (2016). Development of a critical appraisal tool to assess the quality of cross-sectional studies (AXIS). *BMJ Open, 6*(12), e011458. https://doi.org/10.1136/bmjopen-2016-011458

Dunn, L. M., & Dunn, D. M. (2007). *Peabody Picture Vocabulary Test* (4th ed.). Pearson.

Dunn, L. M., Dunn, L., M, Whetton, C., & Burley, A. (1997). *The British Picture Vocabulary Scale* (2nd ed.). NFEF-Nelson.

Evans, M. A., Williamson, K., & Pursoo, T. (2008). Preschoolers' attention to print during shared book reading. *Scientific Studies of Reading, 12*(1), 106-129. https://doi.org/10.1080/10888430701773884

Henry, L., & Maclean, M. (2003). Relationships between working memory, expressive vocabulary and arithmetical reasoning in children with and without intellectual disabilities. *Educational and Child Psychology, 20*.

Hooper, S. R., Costa, L.-J., McBee, M., Anderson, K. L., Yerby, D. C., Knuth, S. B., & Childress, A. (2011). Concurrent and longitudinal neuropsychological contributors to written language expression in first and second grade students. *Reading and Writing, 24*(2), 221-252. https://doi.org/10.1007/s11145-010-9263-x

Joseph, R. M., McGrath, L. M., & Tager-Flusberg, H. (2005). Executive dysfunction and its relation to language ability in verbal school-age children with autism. *Developmental Neuropsychology, 27*(3), 361-378. https://doi.org/10.1207/s15326942dn2703_4

Kim, Y. T., Hong, G. H., Kim, K. H., Jang, H. S., & Lee, J. Y. (2009). *Receptive & Expressive Vocabulary Test (REVT)*. Seoul Community Rehabilitation Center.

Laws, G. (2002). Working memory in children and adolescents with Down syndrome: evidence from a colour memory experiment. *Journal of Child Psychology and Psychiatry, 43*(3), 353-364. https://doi.org/10.1111/1469-7610.00026

Lum, J. A. G., Conti-Ramsden, G., Page, D., & Ullman, M. T. (2012). Working, declarative and procedural memory in specific language impairment. *Cortex, 48*(9), 1138-1154. https://doi.org/10.1016/j.cortex.2011.06.001

Malone, S. A., Burgoyne, K., & Hulme, C. (2020). Number knowledge and the approximate number system are two critical foundations for early arithmetic development. *Journal of Educational Psychology, 112*(6), 1167-1182. https://doi.org/10.1037/edu0000426

Meneghetti, C., Toffalini, E., Lanfranchi, S., & Carretti, B. (2020). Path learning in individuals with down syndrome: The floor matrix task and the role of individual visuo-spatial measures. *Frontiers in Human Neuroscience, 14*, 107-107. https://doi.org/10.3389/fnhum.2020.00107

Metcalfe, J. A., & Stratford, B. (1986). Development of perception and cognitive abilities among nonhandicapped children and children with Down Syndrome. *Australia and New Zealand Journal of Developmental Disabilities*, *12*(1), 65-78. https://doi.org/10.3109/13668258609084070

Michas, I. C., & Henry, L. A. (1994). The link between phonological memory and vocabulary acquisition. *British Journal of Developmental Psychology*, *12*(2), 147-163. https://doi.org/10.1111/j.2044-835X.1994.tb00625.x

Montoya, M. F., Susperreguy, M. I., Dinarte, L., Morrison, F. J., San Martín, E., Rojas-Barahona, C. A., & Förster, C. E. (2019). Executive function in Chilean preschool children: Do short-term memory, working memory, and response inhibition contribute differentially to early academic skills? *Early Childhood Research Quarterly*, *46*, 187-200. https://doi.org/10.1016/j.ecresq.2018.02.009

Obeid, R., & Brooks, P. J. (2018). Associations between manual dexterity and language ability in school-age children. *Language, Speech, and Hearing Services in Schools*, *49*(4), 982-994. https://doi.org/10.1044/2018_lshss-17-0124

Palombo, A.-L., & Cuadro, A. (2020). The incidence of visual-motor processes in the acquisition of orthographic representations in Spanish-speaking schoolchildren (La incidencia de los procesos perceptivo-motrices en la adquisición de las representaciones ortográficas en escolares hispanoparlante). *Estudios de psicología*, *41*(3), 490-509. https://doi.org/10.1080/02109395.2020.1794718

Pickering, H. E., Peters, J. L., & Crewther, S. G. (2019). *A systematic review of the relationship between visual memory and vocabulary during childhood (0-12-years)* PROSPERO International Prospective Register of Systematic Reviews. Available from https://www.crd.york.ac.uk/prospero/display_record.php?ID=CRD42019125132

Rasmussen, C., Wyper, K., & Talwar, V. (2009). The relation between theory of mind and executive functions in children with fetal alcohol spectrum disorders. *The Canadian journal of clinical pharmacology = Journal canadien de pharmacologie clinique, 16*, e370-380.

Resing, W. C. M., Bleichrodt, N., Drenth, P. J. D., & Zaal, J. N. (2012). *Revisie Amsterdamse kinder intelligentie test 2 (Revision Amsterdam Child Intelligence Test 2)*. Pearson Assessment and Information.

Séguin, J. R., Parent, S., Tremblay, R. E., & Zelazo, P. D. (2009). Different neurocognitive functions regulating physical aggression and hyperactivity in early childhood. *Journal of Child Psychology and Psychiatry, 50*(6), 679-687. https://doi.org/10.1111/j.1469-7610.2008.02030.x

Seigneuric, A., Ehrlich, M.-F., Oakhill, J., & Yuill, N. (2000). Working memory resources and children's reading comprehension. *Reading and Writing, 13*, 81-103. https://doi.org/10.1023/A:1008088230941

Semel, E., Wiig, W., & Secord, W. (2006). *Clinical Evaluation of Language Fundamentals* (4th Ed. - Australian standardised edition). Pearson Clinical Assessment.

Senese, V. P., Zappullo, I., Baiano, C., Zoccolotti, P., Monaco, M., & Conson, M. (2020). Identifying neuropsychological predictors of drawing skills in elementary school children. *Child Neuropsychology, 26*(3), 345-361. https://doi.org/10.1080/09297049.2019.1651834

Stokes, S. F., Klee, T., Kornisch, M., & Furlong, L. (2017). Visuospatial and verbal short-term memory correlates of vocabulary ability in preschool children. *Journal of Speech, Language, and Hearing Research, 60*(8), 2249-2258. https://doi.org/10.1044/2017_JSLHR-L-16-0285

Studer-Luethi, B., Bauer, C., & Perrig, W. J. (2016). Working memory training in children: Effectiveness depends on temperament. *Memory & Cognition, 44*(2), 171-186. https://doi.org/10.3758/s13421-015-0548-9

van der Graaf, J., Segers, E., & Verhoeven, L. (2016). Scientific reasoning in kindergarten: Cognitive factors in experimentation and evidence evaluation. *Learning and Individual Differences, 49*, 190-200. https://doi.org/10.1016/j.lindif.2016.06.006

Veraksa, A., Bukhalenkova, D., & Kovyazina, M. (2018). Language proficiency in preschool children with different levels of executive function. *Psychology in Russia: State of the Art, 11*, 115-129. https://doi.org/10.11621/pir.2018.0408

Verhoeven, L., Narrain, G., Extra, G., Konak, O. A., & Zerrouk, R. (1995). *Diagnostische Toets Tweetaligheid (DTT) (Diagnostic test of bilingualism)*. CITO.

Verhoeven, L., & Vermeer, A. (2002). *Taaltoets Alle Kinderen (Language test for all children)*. CITO.

Vukovic, R. K., Fuchs, L. S., Geary, D. C., Jordan, N. C., Gersten, R., & Siegler, R. S. (2014). Sources of individual differences in children's understanding of fractions. *Child Development*, *85*(4), 1461-1476. https://doi.org/10.1111/cdev.12218

Vukovic, R. K., & Lesaux, N. K. (2013). The language of mathematics: Investigating the ways language counts for children's mathematical development. *Journal of Experimental Child Psychology*, *115*(2), 227-244. https://doi.org/10.1016/j.jecp.2013.02.002

Wechsler, D. (2011a). *Escala de Inteligencia de Wechsler para Niños IV (WISC IV)*. Paidós.

Wechsler, D. (2011b). *Wechsler Abbreviated Scale of Intelligence - Second Edition (WASI-II)*. Pearson.

Wechsler, D. (2016). *Wechsler Intelligence Scale for Children, Fifth Edition: Australian and New Zealand Standardised Edition (WISC-V A&NZ)*. Pearson.

Weiss, R. H. (1991). *Grundintelligenztest Skala 2- CFT 20 mit Wortschatztest (WS) und Zahlenfolgentest (ZF) (4th ed.)*. Hogrefe.

Williams, A. M., Marks, C. J., & Bialer, I. (1977). Validity of the Peabody Picture Vocabulary Test as a measure of hearing vocabulary in mentally retarded and normal children. *Journal of Speech and Hearing Research, 20*(2), 205-211. https://doi.org/doi:10.1044/jshr.2002.205

Wilson, J., Andrews, G., Hogan, C., Wang, S., & Shum, D. H. K. (2018). Executive function in middle childhood and the relationship with theory of mind. *Developmental Neuropsychology, 43*(3), 163-182. https://doi.org/10.1080/87565641.2018.1440296

Woodcock, R. W., McGrew, K. S., Schrank, F. A., & Mather, N. (2007). *Woodcock–Johnson III normative update*. Riverside.

Yoo, J., & Yim, D. (2018). Relationship among executive functions, vocabulary and reading skills in school-aged children with and without poor vocabulary. *Communication Sciences & Disorders, 23*(3), 570-583. https://doi.org/10.12963/csd.18523
